# FISHtrees 3.0: Tumor Phylogenetics Using a Ploidy Probe

**DOI:** 10.1371/journal.pone.0158569

**Published:** 2016-06-30

**Authors:** E. Michael Gertz, Salim Akhter Chowdhury, Woei-Jyh Lee, Darawalee Wangsa, Kerstin Heselmeyer-Haddad, Thomas Ried, Russell Schwartz, Alejandro A. Schäffer

**Affiliations:** 1 Computational Biology Branch, National Center for Biotechnology Information, U.S. National Institutes of Health, Bethesda, MD, United States of America; 2 Computational Biology Department, Carnegie Mellon University, Pittsburgh, PA, United States of America; 3 Carnegie Mellon/University of Pittsburgh Joint Ph.D. Program in Computational Biology, Pittsburgh, PA, United States of America; 4 Section of Cancer Genomics, Genetics Branch, Center for Cancer Research, National Cancer Institute, U.S. National Institutes of Health, Bethesda, MD, United States of America; 5 Department of Biological Sciences, Carnegie Mellon University, Pittsburgh, PA, United States of America; Mathematical Institute, HUNGARY

## Abstract

Advances in fluorescence in situ hybridization (FISH) make it feasible to detect multiple copy-number changes in hundreds of cells of solid tumors. Studies using FISH, sequencing, and other technologies have revealed substantial intra-tumor heterogeneity. The evolution of subclones in tumors may be modeled by phylogenies. Tumors often harbor aneuploid or polyploid cell populations. Using a FISH probe to estimate changes in ploidy can guide the creation of trees that model changes in ploidy and individual gene copy-number variations. We present FISHtrees 3.0, which implements a ploidy-based tree building method based on mixed integer linear programming (MILP). The ploidy-based modeling in FISHtrees includes a new formulation of the problem of merging trees for changes of a single gene into trees modeling changes in multiple genes and the ploidy. When multiple samples are collected from each patient, varying over time or tumor regions, it is useful to evaluate similarities in tumor progression among the samples. Therefore, we further implemented in FISHtrees 3.0 a new method to build consensus graphs for multiple samples. We validate FISHtrees 3.0 on a simulated data and on FISH data from paired cases of cervical primary and metastatic tumors and on paired breast ductal carcinoma in situ (DCIS) and invasive ductal carcinoma (IDC). Tests on simulated data show improved accuracy of the ploidy-based approach relative to prior ploidyless methods. Tests on real data further demonstrate novel insights these methods offer into tumor progression processes. Trees for DCIS samples are significantly less complex than trees for paired IDC samples. Consensus graphs show substantial divergence among most paired samples from both sets. Low consensus between DCIS and IDC trees may help explain the difficulty in finding biomarkers that predict which DCIS cases are at most risk to progress to IDC. The FISHtrees software is available at ftp://ftp.ncbi.nih.gov/pub/FISHtrees.

## Introduction

### Background

For at least the past forty years, cancer researchers have collected diverse types of evidence supporting Nowell’s theory that cancer progresses by an evolutionary process [1–3]. Basic questions, such as whether the evolution is punctate or gradual [4, 5], remain open. Nonetheless, it is generally agreed that mutations in single genes, changes of copy numbers of genes, genomic rearrangements, and missegregation of chromosomes or their arms drive tumor progression [2, 6]. Case studies of different sections of the same tumor and of multiple single cells from the same tumor have shown that there can be massive *intra-tumor heterogeneity* with respect to which specific genomic aberrations are present [7–12]. Sampling this heterogeneity and understanding how it arose can provide valuable predictive information for prognosis and treatment planning [13–16]. At early stages of cancer, when treatment is most effective, there may be low-proportion subclones that carry mutations that are especially deleterious *per se* or could later lead to resistance to some types of treatment [17].

The term *tumor phylogenetics* refers to the use of tools from combinatorics, statistics, mathematical optimization, and other areas of mathematics to model tumor progression with an evolutionary perspective. In this work, we present the design of new methods for tumor phylogenetics on fluorescence in situ hybridization (FISH) data from single cells of a solid tumor. We also present a publicly available software implementation of the new methods. The area of tumor phylogenetics was reviewed in 2009 [18], just as large-scale sequencing of tumors became feasible, and again in 2015 [19]. Therefore, we limit our introduction to this topic to only a small set of studies that most influenced the present work on tumor phylogenetics for FISH data.

Some early studies in tumor phylogenetics analyzed dozens of tumors of the same general type (e.g., breast cancer, renal cancer) sampled with techniques such as comparative genomic hybridization to detect large-scale copy number changes and cytogenetics to detect chromosomal breakpoints [20–26]. New methods for the problem of inferring joint models from multiple tumors continue to be developed [27, 28]. The foci of these methods are on modeling *inter-tumor heterogeneity* and the typical order (early vs. late) of genomic changes. Different methods for inferring tree and network models from such cross-sectional data were compared in three different simulation studies [29–31]. These studies concluded that phylogenetic trees or networks could identify the predicted early events as those close to the root and could identify subclasses of tumors as those that had aberrations present in one subtree or subnetwork. One of these studies [29], however, concluded that such cross-sectional analysis of multiple tumors could be misleading with regard to orders of mutations in single tumors, favoring inference from intratumor rather than intertumor heterogeneity. A different approach to identifying subclasses of tumors based on sequence data is to look for different patterns of mutations called “signatures” [32] that seek to describe the process by which somatic mutations accumulate in a tumor.

Next generation sequencing of tumors shifted some of the interest in tumor phylogenetics to addressing the subclonal deconvolution problem in a single tumor (e.g., [33, 34]). Such sequence data are typically presented as lists of variants present in part of the tumor, sometimes along with copy number estimates for different chromosomes or chromosomal intervals. From the sequence data, one can estimate the proportion of sequence reads that carry a variant, often called the variant allele frequency (VAF). Inferring the proportion of cells carrying a variant is challenging and can be confounded by copy-number variations. Repeated sampling over time can evaluate whether a VAF is increasing or decreasing and, in principle, monitoring the VAF of driver mutations can inform treatment decisions. However, since many forms of cancer are life-threatening, it is clinically preferable to model the evolution of subclones from one or more samples soon after the cancer is diagnosed. In the past three years, a large number of methods and software packages have been developed to address the problems of 1) identifying subclones that share a set of variants and 2) building evolutionary models of how the identified subclones are related [35–44]. All these methods rely on the infinite sites assumption, which implies that each mutation of interest arises only once during cancer progression. While the infinite sites assumption is plausible for point mutations and insertions/deletions for most tumor types, it is not plausible for copy number changes of single genes or small regions. Indeed, only a few of the methods listed can take into account copy number information (e.g., [42]). A completely different method for inferring subclones from copy number aberrations, based on interpretation of genomic data sets as mixture models, was proposed by Oesper et al. [45].

The algorithmic problem of identifying subclones would be greatly simplified if one could easily sequence many single cells of the same tumor, thus phasing completely any sequence mutations, or copy number changes. There are still many difficulties including identifying passenger mutations within subclones, detecting mutations affecting a low percentage of cells, sequencing error, lack of complete samples, and extinct subclones. Sequencing from single cells of a tumor was shown to be feasible in pioneering work of Navin and colleagues [4] and has been done in a few published studies to date (e.g. [11, 46–48]). Such data are so far in limited supply, though, and most large-scale studies have relied on bulk sequencing of whole tumor biopsies. For example, the widely used TCGA data are based on bulk sequencing.

An older technology that is feasible in single cells is fluorescence in situ hybridization (FISH), a technique for measuring the copy number of genes or chromosomes. It has been established since the early 2000’s that it is feasible to collect FISH data for a few genes on hundreds of single cells from a tumor [8, 11, 49, 50]. Advances in the multiplexing of the color dyes have gradually increased the number of genes that could be assessed by FISH simultaneously. In this work, we use previously published data sets with four gene probe and one chromosome probe [51], and eight gene probes and two chromosome probes [52], respectively. We are working on data sets with up to fifteen probes, but these are unpublished and do not necessarily have paired samples. The use of probes at the centromeres of chromosomes typically not involved in the cancer being studied makes it possible to estimate the overall ploidy of the cell. The decision about which centromere(s) to use is based on a detailed literature search of the cancer type. The breast cancer data set used two centromere probes and in the few cells where they differed, the centromere probes and gene probes were used to arrive at a consensus ploidy estimate systematically [52]. In contrast, ploidy variations are typically ignored in the methods for reconstructing subclones from bulk sequence data.

The earliest work on single-cell tumor phylogenetics from FISH data used such a ploidy estimate and one or two gene-probes [53]. However, this method was tested only in one pilot study with two probes with an implementation customized to the characteristics of that data set. More recent work on single-cell FISH data has made a number of improvements in models and algorithms for inference of copy number phylogenies, but has either analyzed data sets that did not contain a ploidy estimate, or ignored any ploidy estimate that was available and used only copy numbers of localized gene probes for inference [54–58]. Other phylogenetic studies with FISH data used conventional phylogenetic methods that are designed to model species evolution and that are not necessarily suitable for cancer evolution (e.g., [11, 59]).

In our recent work in this area, we implemented a software package, FISHtrees, to construct multi-scale models of single-cell FISH data, incorporating three types of copy number changes: single gene increases or decreases of one copy, single chromosome increases or decreases of one copy, and genome duplications. The incorporation of genome duplications and a ploidy probe are novel in FISHtrees. Chromosome changes could be inferred in data sets that had two probes on the same chromosome (e.g., [52]). Genome duplications could be inferred based on a doubling of copy numbers of all gene probes [56]. These FISHtrees methods and the method of Martins and colleagues [54] are here called *ploidyless* because they do not use centromere probes to estimate the ploidy of each cell and do not consider ploidy increases or decreases that can arise due to missegregation during mitosis. In contrast, methods that use the centromere probes are called *ploidy-based*.

**Fig 1:**
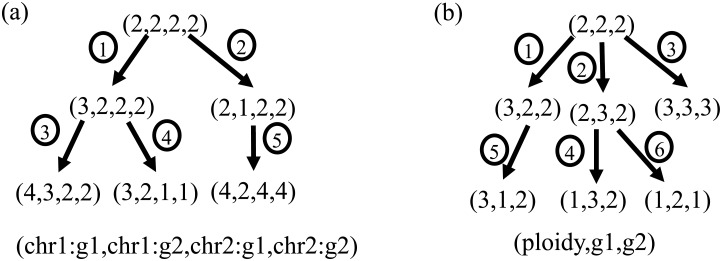
Illustration of ploidyless and ploidy-based trees as might be inferred by the present methods. Circled numbers illustrate the possible mutational event types allowed in each model. (a) Ploidyless tree for two chromosomes with two probes each. Allowed mutation events correspond to (1) gain of a single gene probe, (2) loss of a single gene probe, (3) gain of one copy of (all probes on) a single chromosome, (4) loss of one copy of (all probes on) a single chromosome, and (5) duplication of all measured probes across chromosomes. (b) Ploidy-based tree for a single ploidy probe and two gene probes. Allowed mutation events correspond to (1) increase of ploidy without corresponding gene probe increase, (2) gain of a single gene probe, (3) concurrent gain of ploidy and all gene probes, (4) loss of ploidy without change of gene probe copy number, (5) loss of a single copy of one gene probe, and (6) loss of ploidy concurrent with loss of copy number of all gene probes.

Fig 1 illustrates the distinction between ploidyless and ploidy-based methods. The ploidyless method (panel a) has edges that represent mutations that involve either single genes, all genes on the same chromosome, or a genome duplication event. The chromosome gains or losses and genome duplication events are inferred from changes to the copy numbers of individual genes; there is no specific FISH probe used to infer the copy number of individual chromosomes or the ploidy of the cell. In contrast, the ploidy-based method (panel b) uses an explicit ploidy probe (usually a probe to a centromere) to infer the ploidy of individual cells and to guide tree building.

In this paper, we did a full-scale implementation in FISHtrees of a modified version of the method of Pennington et al. [53]. As explained below, the method is modified partly because experiments on data sets with more than two gene probes revealed flaws in an aspect of the original method involved with merging single-probe tumor progression models. To make this paper self-contained, the full algorithm is presented. Another limitation of the previously implemented tumor phylogenetics methods for FISH data is that they consider only a single tumor sample. Therefore, as part of this study, we developed and implemented a new method for computing “consensus graph models” based on the tree models of two or more samples. The samples could be taken at different times, as was the case for the cervical cancer data set [51], or contemporaneously at different sites, as was the case for the breast cancer data set [52].

### Contributions

The ploidy-based method of Pennington et al. [53] decomposed the problem of modeling tumor progressions for a set of ploidy and gene probes, (*p*, *g*_1_, *g*_2_), for single samples into two subproblems. In this work, we add a third subproblem for datasets that have paired samples. The three subproblems are as follows:

1. For each gene probe *g*_*i*_, compute a tree model using the pairs (*p*, *g*_*i*_) for each cell. We refer to these models as “single-gene trees”. Part of this step is to use expectation maximization (EM) [60] to estimate the probability of each single-gene copy number change and each ploidy change.
2. Merge the models for (*p*, *g*_1_) and (*p*, *g*_2_) into a model for (*p*, *g*_1_, *g*_2_). We implemented a novel generalization to merge all the single-gene trees to obtain a joint tree for all *t* gene probes, but in our experiments here, we focused on merging just two trees as in Pennington et al.
3. (If there are paired samples for each patient) Compute a consensus graph that represents the cell count patterns and transitions shared or not shared between the (*p*, *g*_1_, *g*_2_) models for the two samples.

The prior methods proposed for subproblem 1 used combinatorial and probabilistic algorithms. The methods previously proposed for subproblem 2 used graph theory and mixed integer linear programming (MILP) to solve the MILP instance with the publicly available software glpk (http://www.gnu.org/software/glpk/glpk.html).

In the present paper, we present a new version (3.0) of the software package FISHtrees for single-cell tumor phylogenetics via copy number variation. This software includes a rigorous implementation in C++ of methods along the lines of those proposed in Pennington et al. for subproblems 1 and 2. The new version of FISHtrees includes new methods for the consensus graph problem (subproblem 3 above). The current implementation of the methods for the single-gene trees is similar to the original proposal with some technical changes in the expectation maximization (EM) routine. The current implementation includes a new formulation of tree merging as MILP in the following subsections, designed to address theoretical and practical problems with the original formulation that became apparent as the Pennington et al. MILP was applied to larger data sets now available. We also replaced the older glpk MILP libraries with the newer package SCIP [61] (http://scip.zib.de, results in this study were computed with version 3.02) for solving the MILP instances. SCIP incorporates many techniques for MILP that were not implemented in glpk. The new MILP formulation is presented in a self-contained manner, with occasional comments added to compare to the original MILP formulation of tree merging.

The newly implemented ploidy-based and previously published ploidyless methods are integrated in FISHtrees, so that they can be used together and compared. We show some comparisons of the two types of tumor progression modeling on simulated data. These comparisons show a clear improvement in accuracy of ploidy-based over ploidyless inference when reconstructing tumor phylogenies in the presence of copy number variation at both local and genomic scales.

We also developed a method called “consensus graphs” for comparing tree models derived from multiple samples from the same patient. The consensus graph module can easily be used either in conjunction with our previously implemented ploidyless methods or the newly implemented ploidy-based method because they are all implemented in one package with one set of data structures for internal representations of the data and models. To illustrate the utility of computing consensus graphs, we apply the new methods to a data set on cervical cancer that has paired samples from a primary tumor and a metastasis of the same patient [51]. We also apply the methods to an unusual breast cancer data set in which two samples per patient were collected from samples that had synchronous ductal carcinoma in situ (DCIS) and invasive ductal carcinoma (IDC).

Many genomic studies of DCIS and IDC assumed that in the same patient, the sampled DCIS is necessarily a precursor lesion of the IDC and they sought to identify genomic changes that would enable the transition to invasiveness. For example, Chin and colleagues proposed that telomere crisis is what drives the transition from DCIS to IDC [62]. More recent breast cancer genomic studies, partly reviewed in [2], have questioned whether an observed DCIS is necessarily a precursor to an observed IDC. Our case studies on paired samples show little in common between the cell states of most of the cervical cancer paired samples and, to a lesser degree, a majority of the breast cancer paired samples. Our results suggest that paired primary and metastatic cervical cancers typically diverge from an early precursor. The results are further consistent with the conclusion that, if synchronous DCIS and IDC have a shared precursor, then the two breast malignancies also tend to diverge early in their evolutionary trajectories.

The FISHtrees software is available at ftp://ftp.ncbi.nih.gov/pub/FISHtrees.

## Methods

### Problem Statement

Multi-color FISH technology allows one to count the number of times several probes hybridize to the DNA within an individual cell. For one cell, there is a *configuration* of probes, a vector (*p*, *g*_1_, …, *g*_*n*_) of copy numbers for *n* + 1 probes. The name *p* is distinct because it is a probe that represents ploidy.

A dataset is the record of configuration from all cells counted from a tumor sample. There is no meaning assigned to the chronological order in which cells are analyzed, so the input data are most readily represented as a list of *m* configurations, *C*_*i*_ = (*p*_*i*_, *g*_*i*1_, …, *g*_*in*_) for *i* = 1, …, *m*, and the count *f*_*i*_ of the number of cells in which configuration *C*_*i*_ was observed.

Formally, a dataset *D*_*k*_ is an unordered mapping of configurations {(*p*_*i*_, *g*_*i*1_, …, *g*_*in*_) → *f*_*i*_} for *i* = 1, …, *m*, where *m* is the actual number of distinct configurations observed when counting the cells in sample. In practice, this mapping is supplied as a tab-delimited text file in a format described in the FISHtrees documentation. Analysis can be performed on all probes or a proper nonempty subset of the probes. When a subset of the probes is used, the natural transformation—marginalizing the counts over the unused probes to create a reduced mapping—is performed.

Some studies, such as the cervical cancer and breast cancer studies used here, include more than one sample from some patients. Thus, a study S is a collection of datasets {*D*_1_, …, *D*_ℓ_}, where there may be some further relationships between the data. For instance, there may be paired samples of less advanced/more advanced tumors within the same individual.

For each *D*_*k*_, we build a directed tree *T*_*k*_ = (*V*_*k*_, *E*_*k*_), with vertex set *V*_*k*_ and edge set *E*_*k*_. The vertices *V*_*k*_ represent configurations. All configurations in the dataset *D*_*k*_ are included in the output vertices *V*_*k*_, but occasionally unobserved configurations, known as Steiner nodes, may also be present. Such unobserved configurations represent biologically necessary or likely unobserved states. In particular, the wild-type node—consisting of a count of two for every probe—is always used as the root of the tree, even if no such nodes were observed. In practice, this is a minor issue, as wild-type nodes are observed in most tumor samples. However, there are frequently evolutionarily probable or necessary intermediates not observed in the sample, either by chance or because those intermediates are extinct.

The edges *E*_*k*_ between nodes in the generated tree represent *events*, or mutations that could lead from one configuration of probes to another. Our model restricts the events that can occur to gene gain/loss events, ploidy gain/loss events, and mixed events that represent a failure of segregation during mitosis; more details on the events are presented in the next subsection. While building the trees, we associate each event type with a probability, In the output, we annotate each edge *E*_*k*_ with the probability of its event type. The most desirable tree is one that is consistent with the event model and that has maximum likelihood given the observed data.

To find a desirable tree for dataset *D*_*k*_, we apply a multi-stage process. Our multi-stage solution to this problem does not lead to an explicit objective function for identifying the optimal *T* for a given *D*, although finding appropriate models for this problem is an interesting question for future work.

The multi-stage process starts by creating two single-gene trees: a tree that with nodes (*p*, *g*_*i*1_) for $i=1,\ldots ,\hat{m}$, and a second tree that consists of nodes (*p*, *g*_*i*2_) for $i=1,\ldots ,\tilde{m}$, where $\hat{m}\leq m$ and $\tilde{m}\leq m$ are the number of distinct configurations of nodes when counting only those two probes. A single gene tree is built using an EM algorithm that creates edges and assigns probability to event types based on clonal frequencies derived from the cell counts in dataset *D*_*k*_ The trees generated by the EM algorithms include Steiner nodes, but must only have edges that represent events of permitted type.

We then merge these single gene trees, using the MILP technique described below and detailed in Supplementary material, to produce the output tree *T*_*k*_ = (*V*_*k*_, *E*_*k*_) for dataset *D*_*k*_. The goal of this merge is to find a maximum likelihood tree, where the likelihood of a tree is the product of the probability of all its edges. This objective, along with a term penalizing violation of the event model, is formalized below in the subsection “Tree Merging.” The nodes *V*_*k*_ of the merged tree are of the form (*p*, *g*_1_, *g*_2_), and edges *E*_*k*_ represent permitted events, but at this stage events that may involve a ploidy probe and two gene probes.

In the analysis performed in this manuscript, we proceed at this stage as if each cell is profiled on exactly three probes, one ploidy probe and two non-ploidy probes. One may in principle, apply the joining algorithm not only to trees consisting of a single gene probe, but to trees built from any positive number of gene probes. Thus, given a single-gene tree and a two-gene tree, one may produce a three gene tree, or by extension a tree with any desired number of probes. The FISHtrees code has implementations of two schemes for applying such an extension, as described in Supporting Information, but extension to larger sets of probes is an area of further research.

Finally, one may wish to compare the trees *T*_*ℓ*_ = (*V*_ℓ_, *E*_ℓ_) and *T*_*k*_ = (*V*_*k*_, *E*_*k*_) generated from two different datasets *D*_ℓ_ and *D*_*k*_, taken from the same patient either in a different location or at a different time. For such a comparison, our goal is different. We do not seek to find events that can map one tree to the others, but rather just to determine how similar the trees are. Thus, one generates a *consensus* graph that may include nodes and edges from the single dataset trees. Conceptually, a node or edge is included if it is part of more than one of the single-dataset trees, and below we give multiple formal definitions for when to include nodes and edges in the consensus graph. Consensus trees can be built from any number of single-dataset trees. The test statistics we use to evaluate consensus trees are interpretable for any number of single-data set trees.

### Ploidy-based Modeling

As the name suggests, ploidy-based modeling differs from ploidyless modeling in that the cell count pattern includes the ploidy marker and the allowed single-step transitions take the ploidy into account. In the version we present here, six transitions are allowed between two states (*p*, *g*) and (*p*′, *g*′): the ploidy can increase by 1 (*p*′ = *p* + 1), the gene probe can increase by one copy (*g*′ = *g* + 1), both can increase by 1 (*p*′ = *p* + 1 and *g*′ = *g* + 1); the ploidy can decrease by 1 (*p*′ = *p* − 1); the gene probe can decrease by one copy (*g*′ = *g* − 1); or both can decrease by one (*p*′ = *p* − 1 and *g*′ = *g* − 1). A change in ploidy with no change in the gene probe models a missegregation of chromosomes during mitosis such that the mode of the chromosome number (i.e., the ploidy) changes by one, but the number of copies of the chromosome that happens to contain the gene probe does not change. For multiple gene probes, the tree is built by successively merging trees, according to the rules described below in the section entitled “The Tree Merging Problem”. The rules are complicated and, in fact, constitute an MILP. However, their purpose is simple: to encourage steps that either change the copy number of a single gene, or that change the copy number of all genes in the same direction.

The main steps of the ploidy-based method are summarized in Algorithm 1. More details about the code structure and options for both ploidy-based and ploidyless modeling options are given in the Supporting Information subsection entitled “Code Structure and Main Options”.

To compute maximum-weight branchings, we use the method of Karp [63]. In this context only, the “weight” of a branch is log *p*, a negative number, where *p* is the probability of the type of mutation that this edge represents. Thus, the maximization objective in the branching algorithm favors selecting edges of higher probability. The branching is required to have root (2,2) because that node represents the “normal” state of two copies of every autosome and two copies of the gene probe.

We largely defer discussion of ploidyless modeling to the Supporting Information, as the underlying methods for ploidyless modeling are not novel to this work and are thoroughly described in preceding publications [55–57]. At a high level, the principal differences between the ploidy-based Algorithm 1 and the ploidyless (Algorithm “Top-level view of the parameterized ploidyless method” in S1 Text) include:

- The ploidy-based algorithm uses the ploidy probe *p*, while the ploidyless algorithm does not.
- The ploidy-based algorithm separates the problems of modeling one gene probe and merging the models, while the ploidyless algorithm models all gene probes together throughout.
- The ploidy-based algorithm allows changes in ploidy, but does not model chromosomes, while the ploidyless algorithm models chromosomes and allows changes in ploidy only in the form of genome duplications (GD).

One similarity that facilitates comparison between the methods is that both methods assume that the root of the progression trees must be the all-2 state representing normal diploid ploidy with two copies of every gene probe.

The internal representation of the consensus graph is general enough that it allows different types of transitions involving the ploidy probe and/or one or more gene probes. Therefore, a common code base can be used for consensus tree inference for both ploidy-based and ploidyless modeling. Single gene ploidy-based trees, merged multi-gene ploidy-based trees, ploidyless trees, and consensus networks are all printed in dot format and can be viewed with GraphViz [64].

**Algorithm 1** Top-level view of ploidy-based method, including consensus graph

1: **for** each patient **do**

2:  **for** each sample of that patient **do**

3:   **for** each ordered pair of ploidy *p* and gene probe *g*_*i*_, (*p*, *g*_*i*_) **do**

4:    Construct the directed PossibleGraph with the node set containing all possible copy number states of (*p*, *g*_*i*_) and all edges that represent an allowed mutation

5:    Construct ObservedGraph using function ConnectedNodes from S1 Text.

6:    Initialize the array MutationFrequencies based on the edges in ObservedGraph

7:    Use function OptimalPath from S1 Text to add unobserved (Steiner) nodes and edges to ObservedGraph to make every observed node reachable from the (2,2) root

8:    **while** MutationFrequencies have not converged **do**

9:     *B* ← Maximum-weightBranching(ObservedGraph) with root (2,2)

10:     Use function RandomizedBranching from S1 Text to randomly replace *B* with a new branching *B*′

11:     Update MutationFrequencies based on the edge in *B*′

12:    **end while**

13:    Use the MILP formulation below and SCIP to merge the trees for (*p*, *g*_1_), (*p*, *g*_2_), …

14:   **end for**

15:   **if** there is more than one sample for the patient **then**

16:    Use function Consensus from S1 Text to construct ConsensusGraph containing every joint node and joint edge that appears in at least one merged tree

17:   **end if**

18:  **end for**

19: **end for**

### Algorithms for Ploidy-based Tree Inference

The generation of ploidy-based trees can be decomposed into two subproblems. First, phylogenetic networks are generated for individual gene probes. Second, a set of such networks, corresponding to a common ploidy probe but distinct gene probes are merged into a unified tree model. We provide a high-level description of the major components of each step here, but defer full algorithmic details to the Supporting Information, in the Supplementary Methods.

#### Generating Single-Gene Trees

Three steps are required to generate a single-gene graph. First, since it is frequently the case that not all evolutionary intermediates are present in the observed data, it may be necessary to insert unobserved *Steiner* nodes into the graph. The process for doing so is described in the Supporting Information under the section “Creating a Single-Gene Graph” of the Supplementary Methods. Second, because the graph of all permissible edges is not usually a tree, one must find an optimally weighted tree within the graph, which is the subject of “Selecting a Tree Model from the Single Gene Graph” in the Supplementary Methods. Significantly, which subtree is optimal depends on the assignment of edge weights. The initial tree is built using uniform weights, but a third step, an EM Algorithm is used to optimize the edge weights. As part of the optimization process, the EM Algorithm repeatedly applies the second step of building an optimally weighted tree with its current best estimate of the weights. Full methodological details are provided in the Supporting Information under Supplementary Methods.

#### Tree Merging

We formulate the problem of merging trees on distinct probe sets as an MILP, as in the prior work [53], though with significant modifications as the prior work did not model ploidy changes. The approach sets up an MILP for which feasible assignments of variables correspond to distinct possible trees on the unified probe set across all considered single-gene trees. An objective function is defined that rewards the use of all nodes in a low-weight tree, weakly penalizes the introduction of unobserved nodes, but strongly penalizes the introduction of nodes that contradict the ploidy number measured by the ploidy probe.

All *m* nodes in input tree *A* are assigned an index *i* = 1, …, *m*, and all *n* nodes in input tree *B* are assigned an index *j* = 1, …, *n*. For convenience, for both trees the root is assigned index one. The output of the merging algorithm is some collection of pairs {(*i*, *j*)} which are nodes in the merged tree, and edges connecting these pairs ultimately to the root (1, 1) of the merged tree. The strategy in defining the MILP is to create collections (*a*_*ij*_), (*b*_*ij*_) and (*c*_*ij*_) of binary variables that indicate the existence of an edge between (*i*, *j*) and a parent in the merged tree. The rule *c*_11_ = 1 is the sole exceptional case; we act as if there is an edge into (1, 1), though (1, 1) is the root and so has no parent. We then generate a model so that (*i*, *j*) is in the merged tree if and only if *a*_*ij*_ + *b*_*ij*_ + *c*_*ij*_ ≠ 0.

The full model is summarized as follows.

#### Constants

$$\begin{array}{cc} p_{ij} & \text{observed}\;\text{frequency}\;\text{of}\;\left(i,j\right)\text{;}\;\text{can}\;\text{be}\;\text{zero}\\ w_{k}^{T} & \text{weight}\;\text{of}\;\text{the}\;\text{edge}\;\text{into}\;\text{the}\;\text{node}\;k\;\text{of}\;\text{the}\;\text{input}\;\text{tree}\;T\in \{A,B\}\\ \rho & \text{ploidy}\;\text{mismatch}\;\text{penalty}\\ \sigma & \text{node}\;\text{frequency}\;\text{mismatch}\;\text{penalty}\\ \tau & \text{missing}\;\text{data}\;\text{penalty} \end{array}$$

#### Variables

$$\begin{array}{cc} a_{ij} & \text{presence}\;\text{of}\;\text{edge}\;\text{into}\;\left(i,j\right)\;\text{derived}\;\text{from}\;\text{tree}\;\text{A}\;\text{(binary)}\\ b_{ij} & \text{presence}\;\text{of}\;\text{edge}\;\text{into}\;\left(i,j\right)\;\text{derived}\;\text{from}\;\text{tree}\;\text{B}\;\text{(binary)}\\ c_{ij} & \text{presence}\;\text{of}\;\text{edge}\;\text{into}\;\left(i,j\right)\;\text{derived}\;\text{from}\;\text{both}\;\text{trees}\;\text{A}\;\text{and}\;\text{B}\;\text{(binary)}\\ q_{ij} & \text{frequency}\;\text{of}\;\text{node}\;\left(i,j\right)\;\text{in}\;\text{the}\;\text{joint}\;\text{tree}\;\text{(continuous)} \end{array}$$

#### Simple bounds

$$\;0\leq a_{ij}\leq 1\text{,}\;0\leq b_{ij}\leq 1\text{,}\;0\leq c_{ij}\leq 1\text{,}\;\text{and}\;0\leq q_{ij}\leq 1\text{.}$$

#### Fixed Variables

$$\;\text{For}\;i\neq 1\;\text{and}\;j\neq 1\text{,}\;a_{1j}=c_{1j}=0\;\text{and}\;a_{i1}=c_{i1}=0\text{.}\;a_{11}=b_{11}=0\;\text{and}\;c_{11}=1\text{.}\;$$

#### Nodes have at most one parent

$$a_{ij}+b_{ij}+c_{ij}\leq 1$$

#### A joint edge must have a parent

Let *k* be the parent of *i* in tree *A*, and let ℓ be the parent of *j* in tree *B*.

$$\begin{array}{ll} a_{ij}\leq a_{kj}+b_{kj}+c_{kj} & \text{for }i\neq 1\\ b_{ij}\leq a_{il}+b_{il}+c_{il} & \text{for }j\neq 1;\text{ and}\\ c_{ij}\leq a_{kl}+b_{kl}+c_{kl} & \text{for }(i,j)\neq (1,1). \end{array}$$

#### Only nodes in the merged tree have nonzero frequency

$$q_{ij}\leq a_{ij}+b_{ij}+c_{ij}$$

#### Marginal sums in the joint tree match the observations

$$\;\sum_{j}q_{ij}=\sum_{j}p_{ij}\text{;}\;\text{and}\;\sum_{i}q_{ij}=\sum_{i}p_{ij}\;$$

#### All edges of trees *A* and *B* are used

$$\begin{array}{cc} \sum_{\ell =1}^{n}\left(a_{i\ell }+c_{i\ell }\right)\geq 1, & \text{for}\;i=2,\ldots ,m.\\ \sum_{k=1}^{m}\left(b_{kj}+c_{kj}\right)\geq 1, & \text{for}\;j=2,\ldots ,n\text{.} \end{array}$$

#### Objective Function

∑_*ij*_ *f*_1_ + *f*_2_ + *f*_3_ + *f*_4_

Tree weight is minimized.

$$\begin{array}{c} f_{1}=w_{i}^{A}a_{ij}+w_{j}^{B}b_{ij}+\left(w_{i}^{A}+w_{j}^{B}\right)c_{ij} \end{array}$$

Weight of ploidy-mismatched nodes is penalized.

$$\begin{array}{c} f_{2}=\rho \left(a_{ij}+b_{ij}+c_{ij}\right) \end{array}$$

Mismatches between observed and model frequencies are minimized.

$$\begin{array}{c} f_{3}=\sigma \left|p_{ij}-q_{ij}\right| \end{array}$$

Non-existence of observed nodes is penalized.

$$\begin{array}{c} f_{4}=\tau \left(1-a_{ij}+b_{ij}+c_{ij}\right) \end{array}$$

By default, penalty parameters of the objective function are *ρ* = 1000, *σ* = 100, and *τ* = 100.

The MILP we define is always feasible and thus always produces a solution. Moreover, every feasible point of the program is a tree. Full methodological details, expanded rationales, and proofs are described in the Supporting Information under “The Tree Merging Problem.”

#### Consensus Graph Generation

We implemented a new functionality for generating a consensus graph from a set of trees derived from distinct data sets assayed on a common set of probes. Consensus graph inference is useful for summarizing common features of a set of trees. Such analysis is often valuable for interpretation of tumor data sets, as high patient-to-patient intertumor heterogeneity can make it difficult to identify common features of a tree set manually.

At a high level, consensus tree generation proceeds by sequentially identifying shared nodes between trees and adding those shared nodes and their paths back to the root to a growing consensus graph. The key algorithmic challenge is efficiently identifying paths from shared nodes back to the common root through shared paths in order to avoid exhausting searches of possible paths at each node addition. Detailed pseudocode is provided in the Supporting Information under “Consensus Graph Generation.”

### Input Data

#### Simulated Data

To test the ability of the ploidy-based code to estimate accurately the rates of genetic changes, we randomly generated trees that simulate tumor data under a model of copy number evolution. The algorithm for data simulation is described in detail in the Supporting Information under “Simulated Data” within the subsection entitled “Input Data”. It is similar to the method described less formally in the Supplementary Material of [57]. We specifically generated three data sets, each using two gene probes and one ploidy probe, in order to simplify the comparisons between ploidy-based and ploidyless methods. The data sets differed in the rates with which mutation events were generated, as described in Results. The specific mutation rates for each data set are provided in the columns labeled “Actual” in Table 1. For each such data set, we generated 100 random trees.

**Table 1:** Actual and predicted parameters for the ploidy-based method.

Change	Dataset 1		Dataset 2		Dataset 3	
	Actual	Predicted	Actual	Predicted	Actual	Predicted
(-1,-1,-1)	0.0147	0.00392	0.0177	0.00695	0.0123	0.00517
(-1,0,0)	0.00139	0.000185	0.00168	0.0017	0.000929	0.000954
(0,-1,0)	0.298	0.257	0.203	0.144	0.196	0.15
(0,0,-1)	0.0355	0.0404	0.0502	0.056	0.187	0.144
(0,0,1)	0.379	0.501	0.483	0.619	0.211	0.262
(0,1,0)	0.0294	0.0217	0.0228	0.0181	0.224	0.309
(1,0,0)	0.0161	0.00682	0.0286	0.014	0.0213	0.0262
(1,1,1)	0.226	0.17	0.193	0.141	0.147	0.104

#### Real Tumor Data

We applied our methods on several real tumor data sets. Here, we focus our experiments on data sets for cervical cancer (CC) [51] and breast cancer (BC) [52] because these two data sets contain paired samples from the same patients. Data sets that have paired samples lead to a more elaborate modeling problem than data sets that have only one sample per patient because it is of interest to compare and contrast the two models for each sample in a sample pair.

For each sample, 100–250 single cells were analyzed and the copy numbers of up to eight gene probes *g*_1_, *g*_2_, …, *g*_*t*_ were recorded. The copy numbers of one or two centromere probes *p*_1_, *p*_2_ are also recorded as a proxy for the ploidy of the cell. The centromere probes were selected from autosomes that are typically not gained or lost in that tumor type, so that the centromere probes would be more likely to have the same count as most autosomes.

The cervical cancer dataset contains 16 patients whose tumors metastasized. For these patients, there is one sample of the primary tumor and a chronologically later sample of the metastasis. Due to the time spacing in the study design, it is of interest to ask how copy number changes evolve in the metastasis of each pair. The gene probes are *LAMP3*, *PROX1*, *PRKAA1*, and *CCND1*. These four genes are all oncogenes, meaning that they tend to gain copy numbers as the tumor progresses. All four genes are on different chromosomes. In the CC data set one centromere probe was used a proxy for the ploidy. We sometimes use the “cell count pattern” to mean an ordered list of (ploidy probe count, *LAMP3* count, *PROX1* count, *PRKAA1* count, *CCND1* count) observed in at least one cell of a sample. For example, the cell count pattern (2, 3, 4, 2, 2) means ploidy of 2, 3 copies of *LAMP3*, 4 copies of *PROX1*, 2 copies of *PRKAA1*, and 2 copies of *CCND1*. When we consider a subset of probes, the term cell count pattern refers to only those dimensions of the tuple for the probes being considered. More details about input data and cell count patterns can be found in the Supporting Information subsection entitled “Input Data”.

In the breast cancer dataset, there are 13 pairs of samples of a ductal carcinoma in situ (DCIS) and an invasive ductal carcinoma (IDC) that were sampled *synchronously* for each patient. Because the two samples were taken at the same time in this study design, it is of interest to ask what early copy number changes are shared between the DCIS and IDC of the same patient. The gene probes are five oncogenes (*COX2*, *MYC*, *HER2*, *CCND1*, and *ZNF17*), which tend to be gained as the tumor progresses, and three tumor suppressor genes (*DBC2*, *CDH1*, and *P53*), which tend to be lost as the tumor progresses. *DBC2* and *MYC* both reside on human chromosome 8, *HER2* and *P53* both reside on human chromosome 17, and the other four genes are on different chromosomes. In the BC data set, two centromere probes were combined with the gene probes to infer an estimate of the ploidy as described previously [52].

### Method Validation

We assess accuracy of the methods by a series of measures designed to assess accuracy of parameter inferences, inferred tree topologies, and measures of tree weight that serve as proxies for model likelihood. We summarize the measures here. Full methodological details are provided in the Supporting Information under “Method Validation”.

We first assessed reconstruction quality by accuracy of parameter estimates on simulated datasets, using the following statistic:

$$W=\sum_{i}\frac{{\left(P_{i}-A_{i}\right)}^{2}}{A_{i}},$$

Here, *P*_*i*_ correspond to predicted values and *A*_*i*_ actual values for each mutation type. The value of *W* is a variant of a *χ*^2^ statistic. To assess whether differences in accuracy were significant between ploidy-based and ploidyless methods, we further applied a Wilcoxon paired test to the parameter values inferred by mutation type across all 100 simulated trees in each data set.

To assess accuracies of tree topologies computed by FISHtrees, called here *generated trees*, to the *simulated* trees, we used a minor variant of the bipartition measure as in [56], generalized from [65]. The bipartition measure defines a “reconstruction error”, *R*, that can range from 0% (best) to 100% (worst) based on how similar are the bipartitions of trees obtained by removing single edges. *R* is defined as follows:

$$R=(1-\frac{M}{|T|\times (|P_{s}|+|P_{g}|)-M})\times 100$$

where *P*_*s*_ and *P*_*g*_ are bipartitions of the simulated and generated trees and *M* is a maximum matching of node sets between them.

We further assess tree inference quality by total tree weight summed across edges, where each edge weight takes the form −log(*p*_*i*_), where *p*_*i*_ is the probability of an edge of type *i*. The total weight of a tree is interpretable as a simple log likelihood function for the full tree. For the ploidy-based method, these edge weights are inferred from the observed frequency of edges of each type. For ploidyless trees, maximum-likelihood edge weights are inferred by a separate expectation-maximization step, as described in the Supporting Information.

## Results

We developed and released a new version (3.0) of the FISHtrees software, implementing the ploidy-based method and consensus graph generation. The FISHtrees software is available at ftp://ftp.ncbi.nih.gov/pub/FISHtrees.

### Comparison of the Ploidyless and Ploidy-Based Methods on Simulated Data

#### Assessment by Parameter Accuracy

We first assessed accuracy of the methods using simulated data. Table 1 shows for the three datasets the correspondence between the actual parameter values and the values derived from FISHtrees output. Not surprisingly, the actual distribution of edges among the simulated trees differs somewhat from the parameters input to the simulation code. Simulated trees, by design, can only contain edges of the types listed in the “Change” column. FISHtrees can sometimes produce additional edge types. These edges were interpreted as changes in ploidy affecting all probes, followed by the necessary number of single gene gains or losses. For instance, a vector of copy number changes of the form (1, 1, 0) would be interpreted as the vector (1, 1, 1) (i.e., simultaneous gain of all probes by ploidy increase) followed by the vector (0, 0, −1) (i.e., localized loss of the third probe). Edges for which the parent or child has mismatched ploidy would be simply ignored, but no such edges occurred in these simulations.

We further assessed the accuracy of these inferences, in comparison to those of the ploidyless method, as assessed by the *W* statistic, defined above in the subsection on Method Validation. For this statistic, small values correspond to high accuracy. We found *W* values of 0.076 (ploidy-based) versus 0.78 (ploidyless) for dataset 1, 0.085 (ploidy-based) versus 0.65 (ploidyless) for dataset 2, and 0.083 (ploidy-based) versus 1.1 (ploidyless) for dataset 3. The results show the ploidy-based method to be substantially better at estimating the input parameters.

#### Assessment by Tree Reconstruction Accuracy

Table 2 shows reconstruction errors, as assessed by the reconstruction error measure *R*, for the three simulated datasets. The Table shows that the reconstruction error is low (11–15%) for both the ploidy-based and the ploidyless methods. We showed previously that the reconstruction error is much higher if one uses phylogenetic methods, such as neighbor joining, that are not designed for cancer data [56]. Although the reconstruction errors for both methods are similar, the number of simulated samples (100) is large enough that we can compare the reconstruction errors statistically by a paired Wilcoxon test for each dataset of simulation parameters described above. For datasets 2 and 3, the reconstruction error is significantly less for ploidyless than ploidy-based meaning that ploidyless gave superior accuracy on datasets 2 and 3. They were not significantly distinguishable on simulated dataset 1 (Table 2).

**Table 2:** Reconstruction error for three datasets. P-values were for a Wilcoxon paired test that the distributions are different.

testset	ploidy-based		ploidyless		
	mean	median	mean	median	P-value
Dataset 1	12.62	12.2	12.21	11.8	0.62
Dataset 2	12.63	12	11.21	11.25	0.015
Dataset 3	14.53	14.2	13.03	12.7	0.00046

#### Assessment by Tree Reconstruction Weight (Likelihood)

The ploidyless method tends to generate trees of higher likelihood (corresponding to smaller weight) than does the ploidy-based method (Table 3). This trend is confirmed by a Wilcoxon paired test comparing the weights generated by the ploidy-based and ploidyless method on the same tree.

**Table 3:** Weights generated by the ploidy-based and ploidyless method. P-values were generated using a two-sided paired Wilcoxon test.

testset	ploidy-based		ploidyless		
	mean	median	mean	median	P-value
Dataset 1	57.7	50.5	53.4	49	4.5e-04
Dataset 2	54.6	48.9	48.7	46	4.3e-04
Dataset 3	79.6	69.7	70.8	63.6	1.7e-07

Interestingly, the tendency of the ploidy-based trees to have higher weight is explained by the fact that these trees tend to have more edges. The average weight of each edge in the tree (a quantity equal to the Shannon entropy of the parameters), tends to be significantly less for the ploidy-based trees than the ploidyless trees; see Table 4.

**Table 4:** Average edge weights (entropy) of the generated trees. P-values were generated using a two-sided paired Wilcoxon test.

testset	ploidy-based		ploidyless		
	mean	median	mean	median	P-value
Dataset 1	1.53	1.52	1.64	1.62	7.2e-07
Dataset 2	1.46	1.48	1.58	1.59	2.2e-05
Dataset 3	1.78	1.75	1.94	1.99	2.5e-08

The ploidyless method generates trees solely by minimizing the weight and hence maximizing the likelihood of the tree, adding Steiner nodes implicitly only when they are necessary to connect observed nodes. The ploidy-based method has additional constraints based on the marginal frequencies of the data for the single-gene probes. These constraints oblige it to add additional nodes, and consequently additional edges. Table 5 shows that the average number of unobserved (Steiner) nodes in the generated tree is significantly larger for the ploidy-based method; both methods included all the observed nodes in the trees. That adding these nodes tends to be obligatory for the ploidy-based method was confirmed by running a modified version of FISHtrees that heavily penalized the existence of each non-observed node. The data corresponding to Table 3 did not change substantially.

**Table 5:** Average fraction of unobserved (Steiner) nodes in the generated trees. P-values were generated using a two-sided paired Wilcoxon test.

testset	ploidy-based		ploidyless		
	mean	median	mean	median	P-value
Dataset 1	0.106	0.1	0.0247	0	1.0e-13
Dataset 2	0.124	0.115	0.0289	0.0247	2.4e-14
Dataset 3	0.123	0.127	0.0237	0	6.2e-15

By adding additional nodes and edges to the tree, the ploidy-based method tends to produce more accurate parameter estimates than does the ploidyless method. Analysis described below shows that trees computed on real tumor data typically contain many Steiner nodes. Therefore, the percentages of Steiner nodes produced by the ploidy-based method on the simulated data (Table 5, left columns) are realistic.

Combining the information in Tables 3, 4 and 5, we infer that the ploidy-based method achieves better reconstruction accuracy partly by including more Steiner nodes and the extra Steiner nodes lead to trees of higher total weight, even though the individual edges do not have higher weight on average. Parsimony methods can be expected to be biased towards slightly underestimating true tree weight; these results are consistent with the conclusion that the additional constraints provided by ploidy data make it possible to reduce this bias towards underestimation.

### Application to Real Human Cancer Data

We conducted a series of experiments to evaluate the ploidy-based method on the BC and CC data sets. First, we generated single-gene trees for BC and CC for each gene and its corresponding ploidy probe. We evaluated the single-gene trees by comparing the estimated mutation rate parameters to the observed parameter values. Second, we generated merged trees for all gene pairs and evaluated the two-gene trees by comparing the multi-probe cell count states in the trees to the cell count states in the input data. Third, we evaluated the consensus graphs by analyzing what proportion of states were shared between trees for early cancer samples and advanced cancer samples.

#### Illustrative Example of a Two-Gene Tree

**Fig 2:**
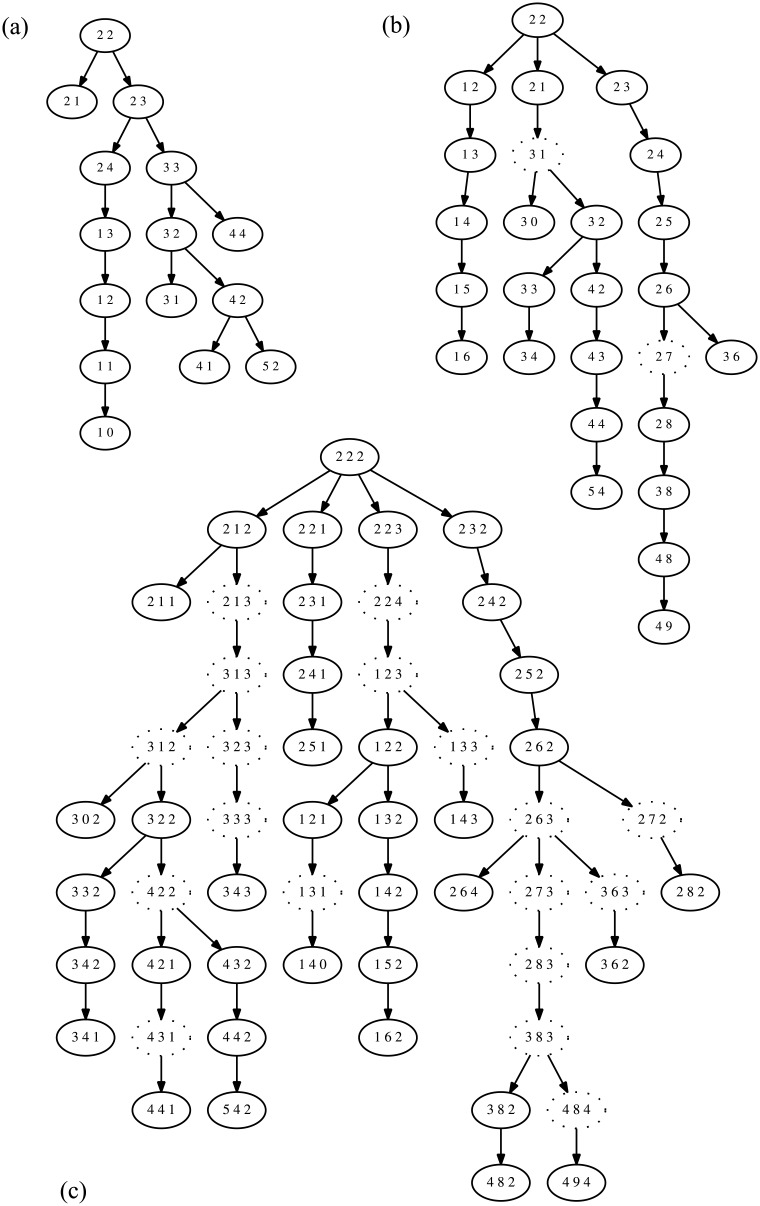
Illustrative example of two single gene trees and a merged tree for a single tumor sample. All trees derive from the BC patient 9 IDC sample. (a) Single-gene tree derived from the *CCND1* gene probe and the ploidy probe. (b) Single-gene tree derived from the *MYC1* gene probe and the ploidy probe. (c) Merged tree for *CCND1* and *MYC* derived from the single-gene trees of (a) and (b).

Fig 2 shows examples of two single gene trees and their two-gene merge trees for data from the breast cancer IDC sample from patient 9 assessed on the genes *CCND1* and *MYC*. Fig 2(a) and 2(b) show the single-gene trees for *CCND1* and *MYC*, respectively. In these single-gene trees, the copy-number count of the ploidy probe is shown on the left and the count of the gene probe is shown on the right. Fig 2(c) shows the corresponding merged tree. For the merged tree, the first number in each oval is the copy number of the ploidy probe, the second the copy number of *MYC* and the third the copy number of *CCND1*.

Both the merged tree (Fig 2(c)) and the tree for *MYC* (Fig 2(b)) have Steiner nodes, indicated by dashed ovals. In fact, the presence of Steiner nodes in the tree for *MYC* makes some of the Steiner nodes in the merged tree obligate. For instance, (3, 1, 3) must be an unobserved (Steiner) node, because (3, 1) was unobserved in the tree for *MYC*. Other Steiner nodes are required because, even though a pairing between trees is possible, there were no observations of the joint state. For instance, (4, 9, 4) was observed in the sample, but no permissible parent of (4, 9, 4) was observed. The tree merging algorithm chooses (4, 8, 4) as a parent. Although (4, 8) is in the tree for *MYC* and (4, 4) in the tree for *CCND1*, (4, 8, 4) was never observed. Other Steiner nodes are added as more complicated consequences of the MILP problem formulation. For example, (1, 3, 3) is a Steiner node because two gains of *MYC* are more likely than one loss of *CCND1*. In all of these cases, Steiner nodes were added *by design* as encoded in the MILP objective function, in the anticipation that they would better model unobserved states than would simply joining nodes.

#### Parameter Estimates from the Single-Gene Trees

We used the single-gene trees in paired tests to evaluate whether gain or loss of any specific genes was characteristic of earlier versus later samples. For the CC dataset, we recorded for each of the 16 patients that had two samples (primary and metastatic) the estimated proportion of gains and losses of each gene from the EM algorithm run on each sample. Then, for each gene, we compared proportions of gains between primary versus metastatic samples in a paired Wilcoxon test. Three of the four oncogenes were gained more frequently in the primary samples than in the metastatic samples; the comparisons for *CCND1* (one-sided p < 0.015) and *PRKAA1* (p < 0.026) were nominally significant, while those for *LAMP3* (p < 0.609) and *PROX1* (p < 0.053) were not significant. All p-values in this paragraph are one-sided.

Analogous paired tests for the breast cancer samples, testing either gains for oncogenes or losses for tumor suppressors, showed no significant differences (data not shown). This is consistent with the observation in the original study that the breast cancer sample is rather heterogeneous in the pattern of gains and losses [52]. That study suggested that gains of the oncogene *MYC* characterized some IDC cases. According to the EM-estimated parameters, gain of *MYC* was more frequent in 9/13 IDC samples compared to the paired DCIS and less frequent in 4/13. Although the differences are very large in some individual pairs, the difference was not large enough overall to be significant by the Wilcoxon test (p < 0.11). In the breast cancer cases, the oncogene *CCND1* was gained more frequently in the IDC samples than in the DCIS samples, but again the difference was not significant (p < 0.23). This suggests that the disproportionate gains of *CCND1* in the early cervical cancer samples are specific to the role of *CCND1* in that tumor type.

#### Coverage and Number of Steiner Nodes in Merged Trees

To assess whether the merged trees represent plausible models of tumor progression, we evaluated the correspondence between cell count states present in the trees and cell count states observed in the data in two ways. First, we examined the proportion of observed states present in the merged trees in comparison to that proportion under the prior Pennington et al. formulation of the tree-merging problem. Evaluation of the previous formulation of the merge step revealed that the merged trees failed to include a substantial proportion of the observed states. This observation motivated the new MILP formulation of the tree merging step described in Methods. Due in part to the parallel change from glpk to SCIP, there were two variants of the “old formulation” considered in this comparison. The “baseline” formulation was implemented with SCIP, but not released in FISHtrees, and it follows the MILP formulation in [53], except for a penalty for merging two single-gene nodes with different ploidies. A “many-to-many” formulation was implemented with glpk, and released with FISHtrees version 2.2. The “many-to-many” formulation differed from [53] in that it allowed single-gene nodes to be reused in merges with multiple other nodes. We refer to the new formulation described herein as “current” and it is the default in FISHtrees, version 3.0.

Using the current formulation of merging trees, the generated trees for both the BC dataset and the CC dataset had a mean coverage of exactly 100%, where coverage is defined as the number of observed nodes in the generated tree divided by the total number of observed nodes. In other words, coverage was perfect, with each generated tree including all the observed nodes in the patient data. Note that generated trees may also include Steiner nodes unobserved in the data but inferred to be ancestral to observed nodes.

In contrast, the merged trees from the many-to-many formulation had a mean coverage in the BC data set of 71.1%, with a minimum coverage of 37.5%. For the CC data set, the mean coverage of the many-to-many formulation was 62.3%, with a minimum of 37.7%. The baseline formulation performed even worse; the BC mean coverage was 59.1% and the CC mean coverage was 45.9%. Thus, the new formulation of the MILP solves the problem that observed multi-probe states were not included in the merged trees.

The second evaluation considered the Steiner nodes that are in the trees but represent unobserved states. We noticed that the merged trees contain a non-negligible fraction of Steiner nodes. For the CC dataset, on average, 15.0% (median also 15.0%) of the nodes in the generated trees were unobserved, Steiner nodes. The BC dataset had a slightly larger number of Steiner nodes, with an average 19.4% (median 17.9%) of the generated trees consisting of Steiner nodes.

To evaluate whether such a large percentage of Steiner nodes is reasonable, we performed a sampling experiment on the BC data set as follows. For each sample file, we generated 100 replicates in which the cell count patterns (for the ploidy and all eight genes) were sampled with replacement following the empirical probability distribution in the observed data. Then, for each triple of the ploidy probe and two gene probes, we counted the number of different three-probe states in the observed data and the number of distinct three-probe states in the random replicates. We averaged the proportion of observed states retained per replicate, either over samples or over probe pairs. The average proportion of observed states retained in the random replicates ranged from 0.747 to 0.830 when averaged over the 26 different samples. The average proportion of observed states retained in the random replicates ranged from 0.778 to 0.811 when averaged over the 28 probe pairs. The average over both samples and gene probe pairs was 0.797. This indicates that in samples of the size used in the BC data one may expect that approximately 20% of the states that could be observed are not, and would need to be inferred as Steiner nodes unless they would be at leaves in the tree. A second reason that Steiner nodes are needed is that cells in intermediate states may disappear in the tumor sample due to negative selection. Thus, we conclude that the average of 19.4% of Steiner nodes in the BC two-gene trees is in line with expectations for the data sizes and distributions of cell count patterns found in the data sets examined.

#### Complexity Measures for Merged Trees

We computed several complexity measures on the merged trees on the BC and CC dataset to determine if the trees for DCIS and IDC breast tumors and primary and metastatic CC could be readily distinguished. We also investigated whether there was a notable difference between the primary tumors of individuals whose CC metastasized and those for which it did not progress.

For all patients and all gene pairs, we counted the number of observed cells assigned to a node at a given depth in a tree. From these values, we calculated the expected depth of an observed node in the tree, where the expected depth is the sum of the depth of a node multiplied by the fraction of observations assigned to that node. The all-2 root node is assigned depth zero.

For each of BC and CC, we performed a Wilcoxon paired test over all patient and gene pairs to determine if the trees for early (DCIS or primary) or advanced (IDC or metastatic) cancer had significantly greater expected depth. In the case of CC, the trees for primary (*early*) tumors had significantly (*P* = 8.0^−13^) greater expected depth, whereas for BC the IDC (*advanced*) trees had significantly (*P* = 5.33 × 10^−6^) greater depth. P-values are two-sided, because we did not know *a priori* whether to expect the early or advanced stage to have greater expected depth.

We then investigated two other measures of tree complexity: Shannon Entropy (SE) and Simpson Index (SI). These two standard measures have been used previously to study the complexity of tumor samples [66, 67]. Both SE and SI measure the difference between the parameters inferred from the tree from the uniform distribution, though for SE a larger number indicates that a distribution is closer to uniform, whereas for SI a smaller number indicates a more uniform distribution. The tumor samples for DCIS were significantly less complex than samples from IDC when evaluated by either SE (*P* = 3.2 × 10^−14^) or SI (*P* = 7.7 × 10^−11^). Conversely, the primary CC samples are found to be more complex than the metastatic samples when measured by SE (*P* = 4.8 × 10^−8^) or SI (*P* = 1.8 × 10^−8^). P-values are two-sided.

That the primary tumors in CC had higher expected depth is consistent with the results previously reported for one type of ploidyless FISHtrees analysis [55]. That the more advanced tumors in the breast had higher expected depth is analogous to results for ploidyless trees inferred from recurring and non-recurring prostate cancer samples [68]. The SE and SI measures are consistent with the expected depth measure, suggesting that the metastatic CC samples are less complex than the primary CC samples, but the IDC samples are more complex than the DCIS samples. The contrast suggests that metastasis involves a different evolutionary process than the evolutionary process in primary tumor progression. The lower average depth of the metastatic samples suggests that a) the subclones that have the capability to metastasize are not necessarily those with the most copy number changes, and b) after metastasis takes place new copy number changes arise in cells that did not necessarily accumulate many changes in the primary tumor phase. An alternative explanation is that the four genes selected for analysis in the CC samples are much more important in the primary tumor phase than in metastasis.

To investigate the contribution of individual probe pairs to the difference in expected depth, SE and SI, we performed Wilcoxon paired tests for each of BC and CC and for each probe pair. For BC, none of the tests revealed a significant difference for any pair of genes, even without correcting for multiple testing. For CC, the measure of expected depth for the pairs (*LAMP3*, *PRKAA1*), (*LAMP3*, *PROX1*) and (*PROX1*, *PRKAA1*) had significant 2-sided p-values at a level of *P* < 0.01 after applying a Bonferroni correction for multiple testing over the six gene pairs tested. For CC, each of SE and SI revealed a significant difference in exactly one pair, (*LAMP3*, *PRKAA1*). Though two-sided p-values are reported, each of these significant differences for pairs of genes was in the direction consistent with the result for all probes; for example, the expected depth of the CC primary trees was greater.

Thus, in both BC and CC data sets, the statistical difference in average tree depth is driven by most or all of the genes, not just one or two. The result may suggest that the difference reflects a more generalized change in the process of genetic diversification in these tumors, as opposed to changes in selective pressures on copy numbers of specific driver genes. We also compared the expected depth of trees generated from primary CC tumor data for patients that did progress to metastatic cancer versus those that did not progress, but found no significant differences.

#### Consensus Between Less and More Advanced Cancer

The CC and BC datasets have paired samples, which makes it possible to compare the two tree models in each pair and estimate what cell count patterns existed in the last common ancestor of the two tumor states. In the Supporting Information section Supplementary Results, subsection “Consensus Example”, we show an example of a consensus graph from the CC data.

To evaluate the set of states are shared between the paired samples, we considered three test statistics and two variants of each:

1. the fraction of observed nodes (cell count states) shared by the two trees;
2. the fraction of observed nodes shared by the two trees and reachable from the all-2 root
3. the fraction of observed nodes shared by the two trees and reachable via a shared path from the all-2 root

Each of the test statistics can be unweighted (count of the nodes) or weighted by the proportion of cells with each given cell count pattern. For the main paper, we restrict our analysis to the weighted variant of the shared path test, which empirically appeared to provide the most consistent results patient-to-patient. All variants, however, show qualitatively similar results (see Supporting Information section “Comparison of Progression States via Consensus Graphs”).

**Fig 3:**
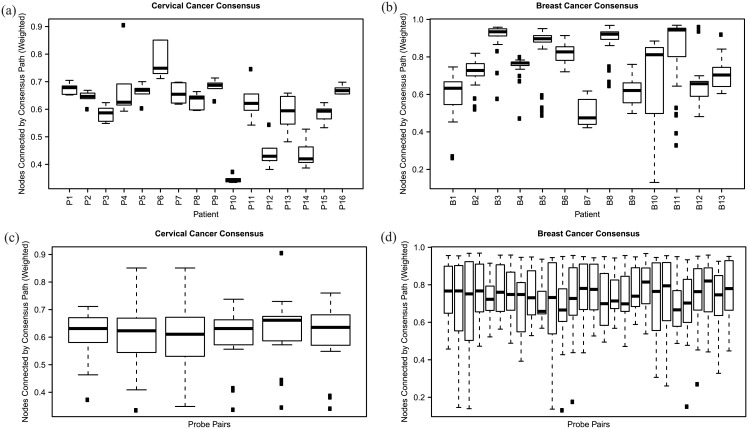
Weighted consensus path values quantifying similarity of trees across progression states. (a) CC weighted consensus path statistics separated by patient, with box-and-whisker plots showing variability by gene pair for each patient. (b) BC weighted consensus path statistics separated by patient, with box-and-whisker plots showing variability by gene pair for each patient. (c) CC weighted consensus path statistics separated by gene pair, with box-and-whisker plots showing variability by patient for each gene pair. (d) BC weighted consensus path statistics separated by gene pair; for each of 28 gene pairs going along the x-axis, there is a box-and-whisker plot showing variability by patient.

Fig 3 shows results from analyses by patient and by gene pair for each cancer type. Fig 3(a) shows weighted consensus path measures patient-by-patient for the CC dataset. The values are low for all patients, never exceeding 0.75, consistent with a model of early divergence of primary and metastatic tumors derived from a common precursor. There is considerable variation patient-to-patient in the measure, however. Variance between gene pairs within single patients is, conversely, relatively low. Fig 3(c) shows these same measures from the point of view of gene pairs, however, showing high consistency in mean values across gene pairs but high variance across patients within each gene pair. The high variance patient-to-patient but low gene pair-to-gene pair indicates that the pattern of early divergence is a generic property of the phylogeny and not a feature of the evolution of specific driver genes.

Fig 3(b) shows a comparable plot of weighted consensus path values for DCIS versus IDC BC data. In this data set, there is a broad range of consensus scores, with six patients having values above 0.8 and seven patients below. The low values in a slight majority of patients are consistent with a notion that DCIS and IDC tumors tend to have early divergence from a common precursor in some patients. The data do not allow us to test definitively whether any particular cases do in fact have a common precursor at all or arise as independent lesions. Again, the statistic shows high variability across patients but low variability across samples within each patient. Fig 3(d) shows the statistic plotted by gene pair, again showing consistent results across gene pairs for BC data but high variance across patients. BC progression from pre-cancer to cancer thus exhibits a qualitatively similar picture to CC progression from primary to metastasis: early divergence, no pronounced driver-gene specificity, but significant variability patient-to-patient in the details.

The Supporting Information, under “Comparison of Progression States via Consensus Graphs,” provides comparable plots for the other variants of the consensus sharing statistic considered. While quantitatively distinct, each shows a qualitatively similar portrait, although with generally higher values for weighted versus unweighted variants and lower values for path versus node statistics.

## Discussion

We described algorithms for the analysis of copy number variation in single cells of a tumor while taking into account an estimate of the ploidy of the cell. All the ploidy-based algorithms are implemented in our software FISHtrees, along with previously described ploidyless algorithms. We tested our methods on simulated data sets and several real tumor sets; for the sake of focus, we presented results on real data only for the CC and BC data sets. These results suggest that taking into account explicit ploidy data improves inference accuracy over prior methods ignoring ploidy counts. These new methods also lead to novel insights into CC and BC data, including identifying statistically significant differences in tree topology between primary and metastatic CC and between DCIS and IDC breast tumors. Our results also support an unconventional model of early divergence between DCIS and IDC in some, but not all breast cancer patients [69], in contrast to the standard notion that IDC is a subsequent progression state of late DCIS. This view may explain why it has been so difficult to find simple biomarkers that predict which cases of DCIS will progress quickly to IDC [70]. Previous analysis by us and others usually simplified the copy number aberrations into three states: gain, normal, and loss. In this ternary model, cell count patterns with 3, 4, 5, etc., copies of a gene would be treated as indistinguishable, while in FISHtrees, we model the exact copy number from 0 to a maximum of 9. This more precise modeling reveals more evolutionary steps and hence more divergence between DCIS and IDC than the ternary model can reveal.

The initial intent of this part of the FISHtrees project was to do a full-scale C++ implementation of the algorithms for single samples outline in [53]. However, initial tests on real data revealed that the original mixed integer linear programming formulation (MILP) of the problem of merging single gene trees would fail to include the majority of observed cell count patterns on some input samples. Therefore, we developed a novel optimization model, revising the constraints and objective function, as described fully in the Methods section entitled “The Tree Merging Problem”. Extensive experiments with the new formulation show that it always includes all observed cell count patterns in the merged tree model, although we did not prove this result theoretically. Our work continues the trend towards using mathematical optimization techniques in tumor phylogenetics [26, 37, 43, 45, 71]. A remaining problem with the tree merging method is that the outcome depends to some extent on the order in which the gene probes are considered because we still merge in one new gene probe at a time.

A variety of tests on simulated data support the hypothesis that making use of ploidy information leads to more accurate tumor phylogeny inferences. An analysis of simulated data suggests that taking ploidy into account leads to more accurate estimates of mutation frequencies. Incorporating ploidy constraints also tends to slightly increase inferred tree weights through increased inferences of unobserved Steiner nodes, providing some correction for an expected systematic underestimation bias of parsimony-based tree inferences. These results provide indirect support for the prior suggestion that taking into account variation in cell ploidy is also likely to improve tumor phylogenetics methods focused on point mutations [38, 39, 42].

The opportunity to analyze the CC and BC datasets that have paired samples motivated us to add the implementation of the consensus graph module of FISHtrees to the newest version (3.0). The consensus graph module can be used in conjunction with either ploidy-based or ploidyless modeling for single samples, but in this account we focused on ploidy-based consensus graphs for the sake of brevity. Our analysis of consensus graphs for paired DCIS and IDC samples suggests that these two states of breast cancer diverge early or may even be separate tumors in some cases. The question of whether and to what extent DCIS and IDC have a common origin has been controversial [2]. Similarly, analysis of consensus graphs of paired primary and metastatic cervical cancer samples suggest that these two tumor states diverge early in their evolutionary histories, consistent with some studies of tumor metastasis in vivo [72–74].

Comparative analysis of the single sample progression trees for DCIS vs. IDC and primary vs. metastasis showed that these two changes of tumor state lead to very different evolutionary trajectories. The ploidy-based trees for the IDC BC samples are statistically significantly deeper than the trees for the corresponding BC DCIS samples (see the Results subsection entitled “Complexity measures for merged trees”). In contrast, the ploidy-based trees for the CC metastatic samples are significantly shallower than the ploidy-based trees for the corresponding CC primary tumor samples; the same observation about tree depth holds qualitatively for ploidyless modeling of the CC data [55]. These properties are robust across genes examined, suggesting that they reflect general properties of the evolutionary process at the two tumor stages, rather than selective biases on specific tumor driver genes. We hypothesize that these differences in tree depth reflect fundamentally different pressures on tumors invading the local tissue as compared to tumor cells growing into full metastases at distant locations. Another possibility is that the contrasting tree depth characteristics are due to as yet unrecognized differences between the evolution of breast cancers and cervical cancers.

This work represents one step in an ongoing process of bringing tumor phylogenetics ever closer to inferring the true complexity of clonal evolution in single tumors. In this study and in our FISHtrees software, we focused on modeling changes in gene copy number, chromosome copy number, and ploidy. Much of the recent work in tumor phylogenetics has been focused on modeling point mutations, since that has been greatly facilitated by next generation sequencing. Modeling point mutations genome-wide is greatly complicated by the large number of genes, but one may be able to use bulk sequencing since the infinite sites assumption that any (point) mutation cannot occur twice in a tumor’s history is plausible. Our studies of copy number variation strongly suggest that the same gene copy number changes can occur multiple times in the evolution of a tumor. Therefore, single-cell sequencing may prove very valuable in modeling copy number evolution if data quality and cost make it feasible to reconstruct accurate single cell sequences on the scales of cells now typical for FISH data. A major two-part challenge in cancer genomics is to do single cell analysis of both point mutations and copy number in enough cells of a tumor to model jointly both types of genetic changes.

## Supporting Information

S1 Text Supplementary methods and results. The Supporting Information includes additional methodological details and results omitted from the main document for clarity of exposition.
(PDF)
