# Supplementary material for: FISHtrees 3.0: Tumor Phylogenetics Using a Ploidy Probe
Source: PLoS One. 2016 Jun 30;11(6):e0158569. doi: 10.1371/journal.pone.0158569 (PMC4928784; doi:10.1371/journal.pone.0158569)
Supplement: S1 Text — The Supporting Information includes additional methodological details and results omitted from the main document for clarity of exposition. (PDF) [file pone.0158569.s001.pdf]

Supplementary Methods

Code Structure and Main Options

The highest level distinction among the modeling options in FISHtrees is whether or not the ploidy probe p is used. Our FISHtrees package is implemented so that the ploidy-based and ploidyless methods share basic data structures for data input, nodes, edges, and graphs. On the command-line, the user can specify whether to do ploidy-based and/or ploidyless modeling. If the user selects ploidyless modeling, then the user must select exactly one of four methods for that part:

1. An exact method for finding minimum cost unweighted trees using only changes of copy number in single genes (denoted as SD events) [1];

2. A heuristic method for efficiently finding minimum cost unweighted trees using only changes of copy number in single genes (SD events) [1];

3. A multiscale method for finding unweighted trees using changes of copy number in single genes (SD), changes in copy number of single chromosomes (denoted as CD), and whole genome duplications (denoted as GD) [2];

4. A multiscale method for finding weighted trees using changes of copy number in single genes (SD), changes in copy number of single chromosomes (denoted as CD), and whole genome duplications (denoted as GD) [3].

Here, as in [3], "weighted" or "parameterized" modeling means that different changes in the cell count pattern (the vector of copy numbers identifying each cell by its probe counts, see the section on Input Data), interpreted as distinct types or locations of copy number mutation event, have different probabilities of occurring and that the probabilities of these different events are estimated as part of the modeling. Method 1 implements an algorithm that is provably exact but exhibits runtime that is worst-case exponential in the number of cell count patterns. Method 2 is a fast heuristic for the problem, which does not provide any guarantees of optimality but yields runtimes polynomial in the number of cell count patterns. Methods 3 and 4 include optimality guarantees for some steps and heuristics for other steps, so they are partly exact and partly heuristic. Since our prior work established that option 4 gives the best models among the ploidyless methods [3] and we previously described all the ploidyless methods in detail, we give only a summary of method 4, among the ploidyless methods, below. By summarizing the ploidyless method 4, we can make the comparison to one ploidy-based method self-contained.

The top level of the parameterized ploidyless method is summarized in Algorithm S1. This is a generalization of the pseudocode entitled EstimateParameters in [3] to allow here for multiple samples per patient and to include the construction of a consensus network. The ploidyless pseudocode here uses nomenclature and notation like that of the ploidy-based Algorithm 1 of the main

paper for easier comparison, whereas in [3] the notation was tailored to describe the procedures at the next layer of the ploidyless method.

Algorithm S1 Top-level view of the parameterized ploidyless method, including consensus graph

```
for each patient do
    for each sample of that patient do
        for all gene probes g_1, g_2, ... do
            Initialize the array MutationFrequencies to be uniform
            Let N be the set of observed nodes
            Construct a tree T to connect N and possibly including Steiner nodes, based on edge weights that are −log of MutationFrequencies
            while MutationFrequencies have not converged do
                Update MutationFrequencies based on the edge types observed in T
                Update T based on the new value of MutationFrequencies
            end while
        end for
        if there is more than one sample for the patient then
            Use Algorithm S8 to construct ConsensusGraph containing every joint node and joint edge that appears in a tree for at least one sample
        end if
    end for
end for
```

Input Data

Data Format Data for phylogenetic inference in this study come in the form of single-cell copy number counts, assessed by fluorescence in situ hybridization (FISH) for cell populations extracted from tumor biopsies. Each data set is assumed to come from a study in which a set of tumors have been assayed on a common set of copy number markers in each of a sample of tumor cells. Depending on the study design, there may be one or more samples per patient in a study.

The input data are represented in one or more files per patient, with each sample in its own file. In the datasets studied here, there are one or two samples per patient. When there are two samples, these represent two stages of tumor progression. However, this assumption is not inherent in our methods; the multiple samples could instead represent multiple physically separate sections of the same tumor [4–6].

Simulated Data Algorithm S2 provides pseudocode for the simulation method, which is based on that of [3]. The simulation creates a tree by starting with a root node and iteratively adding children derived via mutations. The choices

for number of children, which mutations to use, and when to stop depend on random sampling, so that the simulated replicates differ from one another.

Algorithm S2 Generate a simulated tree

```
function SimulateTree(T, p, τ)
    tree ← {(2, 2, 2) → ∅}          ▷ '→' indicates an edge from child to parent
    parents ← {(2, 2, 2)}
    for i ← 1, . . . , 100 do
        edges ← ()                                        ▷ the empty list
        for parent in parents do
            for i ← 1, . . . , 100 do
                break with probability τ.
                Choose k ∈ T based on the probabilities p.
                child ← parent + k
                if IsValidEdge(child → parent) then
                    edges.append(child → parent).
                end if
            end for
        end for
        children ← {e.child | e ∈ edges and e.child ∉ tree }
        break if IsEmpty(children)
        for c ∈ children do
            Randomly choose e ∈ edges so that c = e.child.
            tree ← tree ∪ {e}
        end for
        parents ← children
    end for
end function
```

The inputs to the simulation algorithm are a set T of mutation types, a vector p representing the probabilities of encountering each particular mutation type, and a probability τ that the child-generation algorithm will stop at a given iteration. The use of a parameter τ rather than a fixed number of iterates when generating children causes the number of candidate children generated at each stage to follow a geometric distribution and, in particular, permits there to be nodes with no children at every level of the tree.

Nodes in the generated trees are each defined by the cell count pattern for the ploidy probe, the probe for gene g_1 and the probe for gene g_2. A mutation may be described by a triplet of numbers representing the effects the mutation has on the copy-number counts of each of these probes in moving from the existing parent to the new child. For example, when moving from $(2, 2, 3)$ to $(2, 2, 4)$ the mutation triplet would be $(0, 0, 1)$. Since, for the types of mutations we model here, the copy-number of each type of probe changes by at most one, the numbers in the triplet must each be -1, 0, or 1, and at least one of the numbers must be nonzero. Thus, there are $3^3 - 1 = 26$ possible mutations. For $k \in T$, p_k is the probability with which Algorithm S2 chooses to try to use mutation

k to generate an edge to a child node. Some of the 26 possible combinations are considered to be biologically unreasonable, however. For instance, it is not reasonable that the ploidy count would increase, but the copy number of one of the genes would decrease, in the same mutation. We assign a zero probability to the unreasonable mutation patterns. An example of a mutation that has probability zero is $(1, -1, -1)$.

For the first iteration, we attempt to generate children of the root node $(2, 2, 2)$. At each subsequent iteration, we consider all new nodes from the previous iteration as possible parents, and zero or more edges to child nodes are generated randomly for each possible parent. Tree generation stops when for a given iteration, no new edges are generated, either because the child-generation algorithm randomly chose to generate no candidate edges, or because all of the candidates were rejected, due to rules we describe below. To prevent the possibility of an infinite loop, the algorithm generates trees with a maximum depth of 100, but in practice this limit has little effect as it is rarely reached.

For each individual possible parent, generation of children proceeds as follows. At any iteration, with probability τ the child-generation stops, and with probability $1 - \tau$ an edge to a candidate child node is generated and the algorithm continues to the next iteration. In principle, for each parent, a maximum of 100 (non-distinct) candidate child nodes may be generated, but the chance that the algorithm will not stop before 100 nodes is generated is vanishingly small; for the simulations reported here $\tau = 0.4$.

If an edge to a child node is generated, it is generated by selecting one of mutations in T at random, in proportion to user-specified input probabilities. Often, an edge is generated more than once in this process, and we do not permit multiple edges between nodes in the generated tree. However, all candidate edges are recorded in a list of edges with the multiplicity with which they are generated.

Candidate edges generated in this fashion must be filtered to remove edges with child nodes already in the tree, because adding such edges results in a graph that is not a tree. Edges are also rejected if the corresponding mutation entails a change from a copy number of zero to a nonzero copy number, as there is no simple biological model for such a change. One must also reject edges leading to nodes with impossible copy numbers, negative copy number for any probe or a zero copy number for the ploidy probe. Finally, we also reject edges leading to a node with a copy number above nine, a default maximum copy number for the FISHtrees program. Any observed copy number above this maximum is reduced to the maximum value by FISHtrees for tree inference. Nodes filtered by any of these rules are not replaced. If no children remain, tree generation stops.

Since at each stage, children are generated for each parent in a set of possible parents, it is common for the same copy-number count to be generated via a mutation from more than one possible parent. In such a case, one of the parent-child edges is selected at random, taking into account the multiplicity with which the child was generated for each parent. One way to implement this selection is to create a list of parent-child edges, including an edge in the list each time

it was generated, shuffle the list, and then take the edge representing the first occurrence of each child in the list.

Many trees generated by this algorithm will be too small to simulate cancer genetics; indeed if $\tau = 0.4$, then 40% of the generated trees will have only the root node. We discard any trees that have fewer than 20 nodes or that do not have at least one node with three children. Furthermore, to avoid unreasonably large trees, we discard trees with more than 160 nodes. As a result the simulated trees have on average 33.0 nodes, which is comparable to the average number of distinct copy numbers that were found in real data sets (data not shown). When we write that we ran FISHtrees on 100 replicates, only replicates meeting these quality and size criteria are counted.

Generation of Single Gene Trees

A *single-gene tree* has as its nodes pairs (p, g), where p is a copy-number count of a ploidy probe and g is the copy-number count of a specific gene probe. Despite the data being based on two probes, only one probe is interpreted as the copy number of a relevant gene; the other probe is often to a centromere. We require that the root of the tree be the state (2, 2), i.e., a diploid cell.

As noted in Code Structure and Main Options, edges between nodes in a single-gene tree may represent one of six mutation events: gain of p; loss of p; gain of g; loss of g; simultaneous gain of p and g; or simultaneous loss of p and g. Not all nodes support all types of outgoing edges. For instance, it is not permitted to generate copy-number counts less than zero, or to regain a probe whose copy-number count is zero. Moreover, the default mode of operation for the FISHtrees program forbids ploidy-probe losses from nodes for which the copy-number count of p is greater than two, or gains for nodes at which the count of p is less than two. If an edge between two states is one of the six types listed, and there is no further restriction on taking that edge, we say the edge is *permitted*.

Creating a Single-Gene Graph It is relatively common that the configurations (p, g) observed in the patient cannot all be joined to the $(2, 2)$ root node using only observed nodes and the permitted types of edges. Often, this issue occurs because of the presence of observed configurations having high copy-number in the absence of observations of evolutionarily necessary intermediate configurations. This poses no theoretical problem; cells having the intermediate configurations are presumed to be either unobserved or extinct. However, in these cases, intermediate, unobserved nodes must be added to the graph to allow all nodes to be reached from the root. These unobserved nodes used in tree reconstruction are known as *Steiner nodes*.

We use a recursive heuristic, shown in Algorithm S3, to create a single-gene graph that contains the observed nodes, and for which every node is reachable from the root via a directed path. The algorithm used is similar to the method described in [7] as "Heuristic algorithm for Steiner node inference", or "Algorithm 1," though the description in the previous paper was only at a high

level and does not specify all the details needed to implement the algorithm unambiguously.

The heuristic starts with the collection of observed nodes. We assume that all tumors are rooted at all-diploid normal cell. Typically, the diploid root $(2, 2)$ is among the observed nodes, but if not, as a first step it is added. A graph is then created by linking the current set of nodes by all permitted edges. Because this graph contains the root $(2, 2)$, it may be divided into two parts: a component containing those nodes reachable from the root via nodes in the collection and permitted edges, and a component containing nodes not reachable from the root. Any node (p, q) not currently in the graph, but with children in the component of the graph not reachable from the root, defines an *island*. Specifically, (p, q) is called the *island root*, and the island contains all nodes in the graph that are not reachable from $(2, 2)$, but are descendants of the island root via paths that only pass through nodes currently in the graph. So long as there are nodes not reachable from the root, an island root must exist; every (p, q) has at least one possible parent, and if (p, q) is not reachable from the root, any parent of (p, q) is an island root.

We find an optimal path, in a sense that will be made precise in Algorithm S4, between the component containing the root and an island root defining an island of maximal size. (Several islands may have the same number of nodes). All nodes participating in this optimal path are added to the single-gene graph; after the path is added to the graph, then, all nodes in the island become reachable from the root. Thus, the size of the component of nodes not reachable from the root is strictly reduced. The algorithm recurs, applying the same rules to the new single-gene graph to connect an additional island to the root, until no nodes defining islands remain. A subtle aspect of this recursion is that when nodes are added to the single-gene graph, all permitted edges between the new nodes and nodes already in the graph are also added. In particular, edges that are not in the optimal path are also added to the graph, and these edges may connect other former islands to the root.

Algorithm S3 Create a connected, single-gene graph

Require: Let N be a list of observed nodes, JoinNodes be a function that creates a graph from a list of nodes using all permitted edges, and w be a list of initial edge weights.

function ConnectNodes(N, w)
  Add $(2, 2)$ to N if it is not already present.
  $G \leftarrow$ JoinNodes(N)
  if AllReachableFromRoot(G) **then**
    return G
  else
    path $\leftarrow$ OptimalPath(G, w)
    return ConnectNodes $((N \cup \text{NodesOf(path)}), w)$
  end if
end function

To define what is meant by an optimal path between components, one must assign weights to edges. The weights assigned to edges for the purpose of adding unobserved nodes are computed from initial estimates of the probability of each type of mutation. Better estimates of these parameters will later be calculated using an iterative EM-style algorithm that samples trees formed using nodes and edges from the connected graph.

The initial estimate of each such probability weight is computed by first counting the number of times each of the six types of permissible mutations connects two observed nodes in the single-gene graph via a permitted edge. The initial single-gene graph is rarely a tree because all permitted edges between nodes in the single-gene graph are counted. These counts are normalized to be probabilities p_i. Each possible edge is assigned a weight $-\log p_i$ according to its type. Since, in counting possible edges, only edges between observed nodes were used, the addition of unobserved nodes has no influence on the initial estimate of weight.

Given these edge weights, one may define an optimal path as follows. Let k be the smallest integer such that there is a node v_k that is an island root, and which is connected to the component containing the root by a path with k edges. For the given k, let V' be the set of all nodes that satisfy these criteria, as there is in practice often more than one. Then let V be the subset of V' formed by choosing those nodes that have children in one of the largest islands. Let E be the set of directed edges between the elements of V and their children in the largest islands.

For each edge e in E, we calculate the lowest edge-weight path between the parent node in e and a node in the connected component containing the root. Such a path may be found by standard single-source shortest path algorithms [8]. Then extend this path to one of the largest islands by following the edge e. Of all such paths, we define the lowest-weight paths to be optimal. If there is more than one optimal path, we choose arbitrarily.

Selecting a Tree Model from the Single-Gene Graph In the inner **while** loop of Algorithm 1 of the main paper, we alternate between a) computing a tree that is a minimum-weight branching based on current estimates of MutationFrequencies and b) perturbing the branching to get better estimates of MutationFrequencies. To update the tree model B in steps 10–11 of Algorithm 1, we use a Monte Carlo method, summarized in Algorithm S5, to replace edges in the branching. The combination of the two algorithms follows the framework of expectation-maximization because the branching is updated according to current estimates of the relevant probabilities. In particular, at step 4 of Algorithm S5, the weight of the edge $w \rightarrow v$ in the input to the branching algorithm is -log of the transition probability. We found experimentally that if there are n nodes in Observed Graph, then using NumIterations= $100n^3$ is sufficient to ensure rapid convergence of MutationFrequencies. The expectation-maximization method here is essentially the same as in [7], but the previous work did not report on experiments with the number of edge-replacement steps.

Algorithm S4 Find optimal paths to one or more islands

function OPTIMALPATH(G, w)
  return the empty path **if** ALLREACHABLEFROMROOT(G)
  $H \leftarrow$ REACHABLEFROMROOT(G) ▷ $(2, 2) \in H$.
  $k \leftarrow 1$
  while true do
    $V'' \leftarrow \{$nodes with a path of length k to an ancestor in $H\}$
    $V' \leftarrow \{v \in V'' \mid v$ has a child in $G \setminus H\}$
    break if not ISEMPTY(V')
    $k \leftarrow k + 1$
  end while
  $m \leftarrow \max\{$size(ISLANDEXTENDINGFROM(v)) $\mid v \in V'\}$
  $V \leftarrow \{v \in V' \mid$ size(ISLANDEXTENDINGFROM(v)) $= m\}$
  $E \leftarrow \{(v, c) \mid (v, c)$ is permitted, $v \in V$, and $c \in G \setminus H\}$
  $P \leftarrow \{$PATHAPPEND(p', (v, c)) $\mid p'$ is a minimum weight path from v to $H\}$
  $\ell \leftarrow \min\{$PATHWEIGHT(p, w) $\mid p \in P\}$
  return $\{p \in P \mid \ell =$ PATHWEIGHT(p, w)$\}$
end function

Algorithm S5 Update the tree B derived from OBSERVEDGRAPH and MUTATIONFREQUENCIES via expectation maximization

1: **function** RANDOMIZEBRANCHING(B, OBSERVEDGRAPH, MUTATIONFREQUENCIES)
2:   **for** $i \leftarrow 1$ to NUMITERATIONS **do**
3:     Select one non-root node v uniformly at random
4:     Let $p(v)$ be the existing parent of v
5:     For each possible parent w tabulate the unnormalized probability of selecting w as $f(w) \times$ MUTATIONFREQUENCIES($w \rightarrow v$)
6:     Normalize the probabilities in step 4, so that they sum to 1
7:     Select a possibly new parent w for v in the current tree by sampling from the probabilities in step 6
8:     Let B' be the branching in which $p(v) = w$
9:   **end for**
10:   **return** B'
11: **end function**

The Tree Merging Problem

An MILP is defined by three components: a set of variables, a set of constraints, and an objective function. We consider each in turn.

Variables

Initially, the algorithm for merging single-gene trees is presented with joint frequencies of three probes, two gene probes, gene a and gene b, and a ploidy probe. From these data, we build, as described elsewhere (EM algorithm), two trees: tree A, whose nodes represent copy number counts of gene a and the ploidy probe, and tree B, whose nodes represent copy number counts of gene b and the ploidy probe. In symbols, a typical node in A may be (p_1, x_1) and a typical node in B may be (p_2, x_2), where p_1 and p_2 are the ploidy estimates and x_1, x_2 are the gene probe counts.

We write that nodes (p_1, x_1) and (p_2, x_2) are *ploidy-matched* if $p_1 = p_2$ and are *ploidy-mismatched* $p_1 \neq p_2$. It is logical that two ploidy-matched nodes can be merged to a node (p_1, x_1, x_2). When merging two ploidy-mismatched nodes, the ploidy of the joint node could be either p_1 or p_2. The original formulation did not make a distinction between ploidy-matched and ploidy-mismatched pairs.

The nodes of trees A and B may be, individually, topologically sorted. Thus one may consistently refer to a node in A or B by its index in a list of topologically sorted nodes. Let m be the number of nodes in tree A and n be the number of nodes in tree B. We seek to find a joint graph, more specifically a tree, with nodes of the form (i, j), where i is the index of a node in A and j is the index of a node of B. Only pairs (i, j) that are ploidy-matched represent configurations observable in the patient data. Nevertheless, our notation allows for ploidy-mismatched nodes to be part of the joint graph. In our formulation, existence of joint nodes that merge ploidy-mismatched pairs is highly penalized.

For $0 \leq i \leq m$ and $0 \leq j \leq n$, we introduce binary variables (a_{ij}) that indicate whether the joint tree contains an edge into (i, j) that is derived from an edge in A. Suppose $i \neq 1$, that is to say, i is not the root node of A. If k is the parent of i in A, then $a_{ij} = 1$ if and only if there is an edge from (k, j) to (i, j) in the joint tree. If $i = 1$ then node i has no parent in A and $a_{ij} = 0$. We define the binary variables (b_{ij}) symmetrically. Thus, if $j \neq 1$, $b_{ij} = 1$ if and only if there is an edge from (i, ℓ) to (i, j), where ℓ is the parent of j in B. If $j = 1$, then $b_{ij} = 0$.

The joint graph may also contain edges derived by combining an edge from A with an edge from B. Use of such edges is necessary to avoid introducing ploidy-mismatched joint nodes. Specifically, let k be parent of i in A and ℓ be the parent of j in B, and suppose the joint nodes (i, j) and (k, ℓ) individually have consistent ploidy, but the ploidy of (i, j) is different from the ploidy of (k, ℓ). Then the nodes (k, j) and (i, ℓ) necessarily have mismatched ploidy. Therefore, we prefer to add an edge directly from (k, ℓ) to (i, j), rather than passing through the intermediate nodes (k, j) or (i, ℓ).

For $0 \leq i \leq m$ and $0 \leq j \leq n$, we introduce binary variables (c_{ij}), such

that if k is the parent of i in A and ℓ is the parent of j in B, then $c_{ij} = 1$ if and only if the joint tree contains an edge joining (k, ℓ) to (i, j). If $i = 1$ and $j \neq 1$ or $i \neq 1$ and $j = 1$, then $c_{ij} = 0$. As a sole exceptional case, we define $c_{11} = 1$, even though there is no edge into $(1, 1)$, the root node. The motivation for setting $c_{11} = 1$ is the observation that, if the joint graph is in fact a tree, then for $(i, j) \neq (1, 1)$,

$$a_{ij} + b_{ij} + c_{ij} \geq 1 \tag{1}$$

if and only if (i, j) is in the joint graph. In words, (1) holds for $(i, j) \neq (1, 1)$ because non-root nodes that participate in the joint tree, and only such nodes, are connected by an edge to a parent. Artificially setting $c_{11} = 1$, makes (1) true for any values of (i, j) and thus greatly reduces the need to discuss special cases when specifying the constraints.

In a geometric view, we can think a_i representing the use of horizontal edges (transitioning along the dimension of tree A only), b_i representing the use of vertical edges (transitioning along the dimension of tree B only), and c_i representing the use of diagonal edges (transitioning along the dimension of both single-gene trees simultaneously). The original formulation used only a_i and b_i, allowing only horizontal or vertical edges [7].

Based on a topological order of the nodes of trees A and B, one may define real-valued constants (p_{ij}) so that p_{ij} is the observed frequency of the configuration of probes in the patient data for joint node (i, j), where by definition $p_{ij} = 0$ for ploidy-mismatched nodes. Similarly, we define the variables (q_{ij}) to represent the modeled frequency assigned to node (i, j) in the joint tree by the optimization algorithm.

Constraints

Simple bounds Because (a_{ij}), (b_{ij}) and (c_{ij}) are binary variables, and because (q_{ij}) represent probabilities, it holds that

$$0 \leq a_{ij} \leq 1, \quad 0 \leq b_{ij} \leq 1, \quad 0 \leq c_{ij} \leq 1, \quad \text{and } 0 \leq q_{ij} \leq 1, \tag{2}$$

for all $0 \leq i \leq m$ and $0 \leq j \leq n$.

Fixed variables For $i \neq 1$ and $j \neq 1$,

$$a_{1j} = c_{1j} = 0 \text{ and } a_{i1} = c_{i1} = 0. \tag{3}$$

Moreover, $a_{11} = b_{11} = 0$ and $c_{11} = 1$. Bounds fixing the variables are consistent with, but supersede, the simple bounds (2).

Joint nodes may have at most one parent. For all $0 \leq i \leq m$ and $0 \leq j \leq n$,

$$a_{ij} + b_{ij} + c_{ij} \leq 1. \tag{4}$$

If $a_{ij} + b_{ij} + c_{ij} = 0$, then (i, j) is not in the joint graph.

Joint edges must originate from nodes in the graph.

$$a_{ij} \le a_{kj} + b_{kj} + c_{kj}, \qquad \text{for } j = 1, \ldots, n \text{ and } i \ne 1, \tag{5a}$$
$$b_{ij} \le a_{i\ell} + b_{i\ell} + c_{i\ell}, \qquad \text{for } i = 1, \ldots, m \text{ and } j \ne 1; \text{ and} \tag{5b}$$
$$c_{ij} \le a_{k\ell} + b_{k\ell} + c_{k\ell}, \qquad \text{for } (i, j) \ne (1, 1). \tag{5c}$$

The variables a_{11}, b_{11} and c_{11} are not the left hand side of any constraint in this set. However, these variables have fixed values, with $a_{11} = b_{11} = 0$ and $c_{11} = 1$.

Weight is assigned only to nodes in the graph. For $0 \le i \le m$ and $0 \le j \le n$,

$$q_{ij} \le a_{ij} + b_{ij} + c_{ij}. \tag{6}$$

Marginal sums of observed and modeled frequencies are the same. For, $i \ne 1$, let k be the parent of i in A, and if $j \ne 1$, let ℓ be the parent of j in B. We require that

$$\sum_j q_{ij} = \sum_j p_{ij} \qquad \text{for } i = 1, \ldots, m; \text{ and} \tag{7a}$$
$$\sum_i q_{ij} = \sum_i p_{ij} \qquad \text{for } j = 1, \ldots, n. \tag{7b}$$

Recall that the values (p_{ij}) are constants based on the observed patient data. Thus, the right hand side of these constraints is constant.

This is subtly different from the consistency constraints in the original formulation, which allowed unobserved Steiner nodes to have non-zero weight.

Each edge in trees A and B is used at least once. These constraints are specified in two parts, one part involving (a_{ij}) and one part involving (b_{ij}).

$$\sum_{\ell=1}^{n} (a_{i\ell} + c_{i\ell}) \ge 1, \ \text{ for } i = 2, \ldots, m. \tag{8a}$$
$$\sum_{k=1}^{m} (b_{kj} + c_{kj}) \ge 1, \ \text{ for } j = 2, \ldots, n. \tag{8b}$$

Equation (8a) also holds for $i = 1$, and (8b) holds for $j = 1$, but in each case the constraints only involve fixed variables.

In the original formulation, there was a stricter constraint that each edge in trees A and B should be used exactly once. The stricter constraint is intuitively based on the principle of parsimony. However, we observed experimentally that the stricter constraint led to very small joint trees in which many observed nodes could not be included.

Objective Function

The objective function is built by combining several desired features of an optimal tree. We consider components of the objective intended to favor each such desired feature separately.

The weight of the edges is minimized. The first set of terms in the objective function have the form

$$w_i^A a_{ij} + w_j^B b_{ij} + (w_i^A + w_j^B) c_{ij} \tag{9}$$

Where for $i \neq 1$, $w_i^A a_{ij}$ is the weight in tree A of the edge entering node i. The value of $w_1^A = 0$. The constants w_j^B are defined symmetrically.

Existence of ploidy-mismatched nodes is penalized. There is a penalty term of the form

$$\rho(a_{ij} + b_{ij} + c_{ij}) \tag{10}$$

in the objective function for all nodes (i, j) for which the count of the ploidy probe in node i does not match the count of the ploidy probe in node j. The value of ρ is intended to be large, making the penalty effectively infinite. In practice, we use $\rho = 1000$.

In truth, we would prefer to require

$$a_{ij} + b_{ij} + c_{ij} = 0 \tag{11}$$

for all ploidy-mismatched terms, which would strictly prohibit ploidy mismatches. However, it is not clear that for all possible input trees A and B, each of which was built individually and may have had unobserved nodes added, constraints of the form (11) must be feasible. However, penalty terms of the form (10) may be said to be *exact*, in the sense that if (11) is feasible, then there is a $\hat{\rho}$ so large that (11) is satisfied for all $\rho \geq \hat{\rho}$ [9].

Mismatches between the observed and modeled frequencies are penalized. The penalty is of the form

$$\sigma |p_{ij} - q_{ij}| \tag{12}$$

where a typical value of the penalty parameter $\sigma = 100$. While terms of this form are not linear, there are standard techniques that may be used to transform the absolute value into a linear function using auxiliary variables. [9].

Nonexistence of observed nodes may be penalized. One may add a penalty of the form

$$\tau(1 - a_{ij} + b_{ij} + c_{ij}) \tag{13}$$

for all (i, j) not observed in the original data. Terms of this form encourage the joint tree to use all observed nodes. Some nonlinear functions, such as the relative entropy

$$\sum p_{ij} \log(p_{ij}/q_{ij})$$

force weight (q_{ij}) to be assigned to all nodes for which $p_{ij} \neq 0$, and thus by (6) force (i, j) to be part of the joint graph. Penalty terms of the form (12) do not force existence of observed nodes, but have the advantage of being linear. The penalty terms (13) attempt to partially capture the properties of the nonlinear function.

Currently, we set $\tau = 100$.

The objective function used here is substantially different from that in the original formulation [7].

Properties of the Tree Merging Optimization Problem

Proposition 1. *The optimization problem is feasible.*

Proof. Let $p_i^A = \sum_j p_{ij}$ and $p_j^B = \sum_i p_{ij}$. Consider, then, the point

$$q_{ij} = p_i^A \times p_j^B \tag{14a}$$

$$a_{i1} = 1 \qquad \text{for } i \neq 1 \tag{14b}$$

$$b_{ij} = 1 \qquad \text{for } j \neq 1 \tag{14c}$$

$$c_{11} = 1, \tag{14d}$$

with $a_{ij} = 0$, $b_{ij} = 0$ and $c_{ij} = 0$ for any (i, j) for which the variables were not explicitly set to 1. We claim that, while unlikely to be optimal, the point (14) is feasible, and thus the problem itself is feasible.

The proposed feasible point (14), satisfies all simple bounds (2) and (3) on the variables. Every node, except $(1, 1)$ has exactly one parent. If $i \neq 1$, but $j = 1$, that parent is $(k, 1)$ where k is the parent of i in A. If $j \neq 1$, then that parent is (i, ℓ) where ℓ is the parent of j in B. Thus constraint (4) holds, with equality, for all (i, j).

Because every possible node (i, j) has been included in the graph defined by (14), constraints sets (5) and (6) trivially hold. Moreover, because q_{ij} was defined to be the product distribution of p_i^A and p_j^B, the constraints (7) on the marginal sums hold. Finally, each edge in A is used once, and each edge in B is used m times, and thus more than once, satisfying constraints (8). Thus the point (14) is feasible.

□

Proposition 2. *Every feasible point defines a tree.*

Proof. Because the nodes of A and B are topologically sorted, if k is a parent of i in A, and ℓ is a parent of j in B, the pairs (k, ℓ), (k, j), and (i, ℓ), when considered as indices, are all lexicographically strictly less than (i, j). Thus, any

edge in the joint graph leads to a parent with strictly lexicographically lower indices.

We show by induction that every node (i, j) is either not in the joint graph or is connected by a path to the root. The root, $(1, 1)$, is connected to itself. Suppose that every node lexicographically less than (i, j) is either not in the graph, or is connected to the root. If (i, j) is not in the graph, then the proposition trivially holds. If (i, j) is in the joint graph, then by the constraint (5), it is connected by an edge to a parent node lexicographically less than itself, and the parent node is in the graph. By the inductive hypothesis the parent node is connected to the root, so (i, j) itself is connected to the root.

Moreover, it holds that the joint graph is a directed acyclic graph (DAG). Otherwise, there must be a cycle in the joint graph. However, at least one edge in a nontrivial cycle must connect a child to a parent lexicographically *greater* than itself. As we have determined, edges must connect a child to a parent lexicographically less than itself, so the existence of cycle is a contradiction.

Interestingly, we have shown that the joint graph is a DAG based only on the fact that input graphs A and B may be topologically sorted, i.e., that they are themselves DAGs. Though our formulation only allows nodes in trees A and B to have one parent, one may add additional variables to allow multiple parent edges. However, so long as A and B are DAGs, all edges must connect a child to a lexicographically lesser parent.

Finally, by constraint (4), each node in the joint graph may have at most one parent. Thus the joint graph is a connected DAG for which all nodes have at most one parent. In other words, the joint graph is a tree. □

The Ploidy-Based Method for More Than Two Genes

In this manuscript, we have approached the issue of datasets with more than two gene probes by analyzing pairs of genes from each dataset. It is possible to extend the ploidy-based method to more than two genes, though the most desirable but computationally tractable way to do so remains a topic of further research.

The FISHtrees code provides two schemes for using more than two gene probes. Both these schemes are based on the observation that the result of joining two trees is itself a tree, with all the properties required for input to the tree merging algorithm. Thus, once one has a two-gene tree, one may merge it with a single-gene tree to produce a three-gene tree, and so on.

Currently, the FISHtrees code can perform merges for which the right tree, tree B, is a single-gene tree, though the left tree, A, may represent the data from any number of genes. The simplest method, then, of producing an n gene tree is to merge single gene trees in the order in which the genes are supplied by the user, from left to right. This is the default mode of FISHtrees when there are more than two genes. An alternative, enabled using the command-line switch '`--choose-gene-order`', is to have FISHtrees heuristically reorder the gene list in an attempt to find a low-weight tree.

Formally, the heuristic proceeds as follows. First, reorder the set of n genes so that the gene symbols $\{g_i\}$ for $i = 1, \ldots, n$ are in ascending order lexicographically (according to ASCII character order). The output of the heuristic is not expected to depend greatly on the input order of the genes. The order of the first two genes is arbitrary because the MILP for merging the first two trees is symmetric. Thus, it is convenient to specify that the first two genes be used in lexicographic order. There are other unlikely situations in which ties need to be broken, and it is preferable that these ties not be broken based on the input order of the genes.

Let A_i for $i = 1, \ldots, n$ be a collection of n single-gene trees, generated as described in the section "Generation of Single Gene Trees". Merge all $n(n-1)/2$ pairs of trees (A_i, A_j) where $i < j$. Let (A_k, A_ℓ) be the pair with minimum weight, using lexicographic ordering of the gene names to break ties.

Then, reorder the list of trees A_i for $i = 1, \ldots, n$ to create a list of trees B_j for $j = 1, \ldots, n$ where $B_1 = A_\ell$, $B_2 = A_k$, the genes corresponding to B_j for $j = 3, \ldots, n$ are in lexicographic order. Then, proceed using Algorithm S6.

Algorithm S6 Create a merged tree from several single-gene trees

Require: Let B_j be a list of single-gene trees, let MergeTrees() be an implementation of the tree merging problem, and Weight() be a function for calculating the weight of a tree.

$\mathcal{I} \leftarrow \{1, 2\}$
$T \leftarrow$ MergeTrees(B_1, B_2)
while $\mathcal{I} \neq \{1 \ldots n\}$ **do**
    $S \leftarrow T$
    $w \leftarrow \infty$
    $\mathcal{I}' \leftarrow \mathcal{I}$
    for $j \leftarrow 1, \ldots n$ **do**
        next if $j \in \mathcal{I}$
        $T' \leftarrow$ MergeTrees(S, B_j)
        if Weight$(T') < w$ **then**
            $w \leftarrow$ Weight(T')
            $T \leftarrow T'$
            $\mathcal{I}' \leftarrow \mathcal{I} \cup \{j\}$
        end if
    end for
    $\mathcal{I} \leftarrow \mathcal{I}'$
end while
return T

Consensus Tree Generation

Let trees J and K have nodes representing the copy numbers of the same set of probes, and let each tree be rooted at the same normal diploid copy number count. A node in tree J and tree K is *shared* if it occurs in both trees. A node

is *observed* if it was seen in the patient data used to generate at least one of trees J and K. A node may be shared by trees J and K, but observed only in the data used to generate one of the trees, say J. Such a node is observed and shared, although it was observed in the patient data used to generate K, but was rather inserted into K as a Steiner node by the tree generation algorithm.

We say an observed, shared node j *shares a path* from the root in J and K the root if it meets the following criteria. Let R be a list of nodes, starting at the root and proceeding via edges in J to the node j. Since J is a tree and j is a node of J, the path exists and is unique. Similarly, let S be the path in K connecting the root of K to j. Let R' be the list formed by omitting all unobserved or unshared nodes from R, and let S' be similarly derived from S. If R' and S' contain exactly the same nodes in the same order, then j shares a path from the root in J and K.

Pseudocode for consensus tree generation is shown as Algorithms S7 and S8. The algorithms assume the existence of a routine ObservedSharedNodes that can consult the patient data to determine which nodes shared between two trees were represented in the observations. The algorithms were designed based on the observation that in a tree, each node, except the root, has exactly one parent and that the tree can be specified by listing the edges from child to parent.

Algorithm S7 Find edges in paths common to trees A and B

```
function CommonPaths(A, B)
    parents ← an empty associative array.
    N ← ObservedSharedNodes(A, B)
    for node in N do
        a ← A.parent(node)
        while a ≠ ∅ and a ∉ N do
            a ← A.parent(a)
        end while
        b ← B.parent(node)
        while b ≠ ∅ and b ∉ N do
            b ← B.parent(b)
        end while
        if a ≠ ∅ and a = b then
            parents[node] = a
        end if
    end for
    return parents
end function
```

Algorithm S7 takes each observed shared node and maps it to its nearest observed shared ancestor in A, if any, and its nearest observed shared ancestor in B. If both these ancestors exist and are the same, then it records the (common) ancestor as the parent of the shared node. Algorithm S8 then starts at each observed shared node and follows the path of its observed shared ancestors until

either a node known to be connected to the root via a shared path is found, a node known not be connected to the root is found, or the path ends without finding the root node. Algorithm S8 maintains a growing consensus tree and a growing list of observed shared nodes not connected to the root by a shared path. As soon as it is determined that an observed shared node is connected to, or is not connected to, the root via a shared path, the node is added to one of these two sets. Thus the path extending from each observed shared node to the root only needs to be considered once.

Algorithm S8 Generate a consensus tree

```
function Consensus(A, B)
    N ← ObservedSharedNodes(A, B)
    parents ← CommonPaths(A, B)
    T ← {(2, 2, 2, 2), ∅}                        ▷ root and no edges to start
    S ← ∅                                        ▷ nodes not in the consensus tree
    for node in N do
        ancestor ← node
        while ancestor ∉ T and ancestor ∉ S do
            break unless exists(parents[ancestor])
            ancestor ← parents[ancestor]
        end while
        if ancestor ∈ T then
            while node ∉ T do
                T ← T ∪ {parents[node → node]}   ▷ add edge to newly reached node
                node ← parents[node]
            end while
        else                                     ▷ not connected to root
            while exists(node) and node ∉ S do
                S ← S ∪ {node}
                node ← parents[node]
            end while
        end if
    end for
    return T
end function
```

Method Validation

Assessing Tree Reconstruction Error Let T be the set of nodes common to the generated and simulated trees. Let P_s and P_g represent the sets of non-trivial bipartitions in the simulated and generated trees, respectively, obtained by removing a single edge. Each bipartition is implicitly associated with the edge removed to create the bipartition. Then,

$$R = \left(1 - \frac{M}{|T| \times (|P_s| + |P_g|) - M}\right) \times 100$$

where M is the weight of the maximum matching of bipartitions between the simulated and generated trees as explained below.

To compare simulated trees to generated trees, it is necessary first to remove the Steiner nodes from the generated trees. To reduce the trees with Steiner nodes to trees without Steiner nodes, we performed a compaction of paths as follows. First, all Steiner nodes were removed from the generated tree. The simulated tree by definition contains no Steiner nodes. If a path in the original, generated tree had observed nodes as endpoints but only Steiner nodes as intermediate nodes in the path, an edge directly linking the endpoints was added to the generated tree. Because the generated tree is, in fact, a tree, at most one path can join two observed nodes, and there is no ambiguity in path shrinking.

Next all non-trivial bipartitions of the simulated tree and the generated tree were computed. A weight was assigned to each pairing of a bipartition of the simulated tree with a bipartition of the generated tree based on the number of nodes in common [2]. We note one crucial detail: when computing the number of elements in common between bipartitions $(\mathcal{A}_1, \mathcal{A}_2)$ and $(\mathcal{B}_1, \mathcal{B}_2)$, the maximum of the pairing $(\mathcal{A}_1 \approx \mathcal{B}_1, \mathcal{A}_2 \approx \mathcal{B}_2)$ and $(\mathcal{A}_1 \approx \mathcal{B}_2, \mathcal{A}_2 \approx \mathcal{B}_1)$ was used. The optimal pairing of bipartitions was computed using Munkres' algorithm for maximum weighted matching in bipartite graphs.

Unlike the method described in [2], we did *not* remove nodes that were missing from the generated tree from the simulated tree. Munkres' algorithm was run using a version that does not require a square adjacency matrix (representing the graph of bipartitions). This version of Munkres' algorithm leaves some bipartitions from the larger tree unmatched. This change does not affect the weight of individual matches between bipartitions, but affects the calculation of the reconstruction error. We deemed this change necessary to avoid extreme cases in which small trees are given low reconstruction error; a singleton tree would be a perfect reconstruction in the old scheme. On the actual data, however, the ploidyless method always uses all the observed nodes, and the ploidy-based method usually uses all the nodes (data not shown).

Comparison of Ploidyless and Ploidy-Based Methods by Weight Another way to evaluate the quality of the generated trees is based on the objective function, which apart from penalty terms (which are usually zero) encourages the algorithm to find a tree of minimal weight, while (usually) using all the observed nodes. Finding a tree of minimal weight maximizes the likelihood of the tree given the model. The problem is phrased in terms of minimizing the weight of all edges in the tree, but these weights take the form $-\log(p_i)$, where p_i is the probability of an edge of type i, estimated by counting edges in that specific tree.

For the ploidy-based code, the weights obtained by counting *are exactly* the appropriate weights to use. For the ploidyless method, there are other weight estimates, obtained from the EM method. However, using these probabilities rather than the counted frequencies can only produce weights larger than the

weights obtained by counting. To clarify the distinction, let q_i be the printed frequencies and let n be the number of nodes in the tree. Then, the difference between the two weights is: $n(\sum -p_i \log q_i - \sum -p_i \log p_i)$. This difference is the number of nodes times relative entropy, $\sum p_i \log(p_i/q_i)$. Because relative entropies are always non-negative, it follows that the difference in weights is non-negative and the weights based on the counted frequencies are smaller. Moreover, the ploidyless method does not necessarily print edges involving Steiner nodes, in part because for paths longer than one edge, the order of edges is ambiguous. One may, however, generate optimal Steiner paths using the methods described in Chowdhury et al. [3], and we do so before counting the edges.

Use of Monotonic Ploidy Rule For several reasons, we used a *monotonic ploidy* rule, the rule that configurations with ploidy greater than two could only gain ploidy, and those with ploidy of one could not regain ploidy. Biologically, if survival of clones with a gain or loss of ploidy is favorable, then it is unexpected that reversing such a change would also be favorable. Mathematically, the evolutionary process is modeled by trees, but in general, modelling an evolutonary process without monotonic changes in ploidy involves cycles, which trees cannot have. Practically, omitting this rule resulted in considerably worse performance by multiple criteria, without any evident benefits. We discuss the results of tests performed omitting the monotone ploidy rule in the Supplementary Results section “Comparison with Montonic Ploidy Rule Omitted”.

Supplementary Results

Consensus Example

Figure S1 illustrates the consensus graph for the CC patient P1 for the gene pair *LAMP3* and *CCND1.* Nodes shared by both the primary and metastatic trees are shown as ovals, nodes only in the primary tree are shown as rectangles, and nodes only in the metastatic tree are shown as diamonds. Dotted nodes did not appear among the observations, i.e., are Steiner nodes. A solid directed edge between nodes indicates an edge shared by both trees, whereas dashed directed edges only appear in one tree. It happens, for instance between nodes $(1, 1, 2)$ and $(1, 1, 3)$, that the two trees connect the nodes in opposite directions, due to a predicted preference for gain of *CCND1* in the primary sample and loss of *CCND1* in the metastatic sample. These two directed edges correctly indicate a lack of consensus between the two generated trees.

Overall, Figure S1 paints a picture consistent with the complexity results from “Complexity Measures for Merged Trees” in the main paper. The primary tree is larger and more complex, with many nodes (indicated by rectangles), and consequently many edges, not in consensus with the tree from the metastatic sample. By contrast, the tree generated from the metastatic sample has few nodes (indicated by diamonds) not shared by both trees.

Figure S1: **Consensus graph for CC patient P1, genes *LAMP3* and *CCND1*.** The numeric triple shown in each node indicates the copy number counts of the triple (ploidy, *LAMP3*, *CCND1)*.

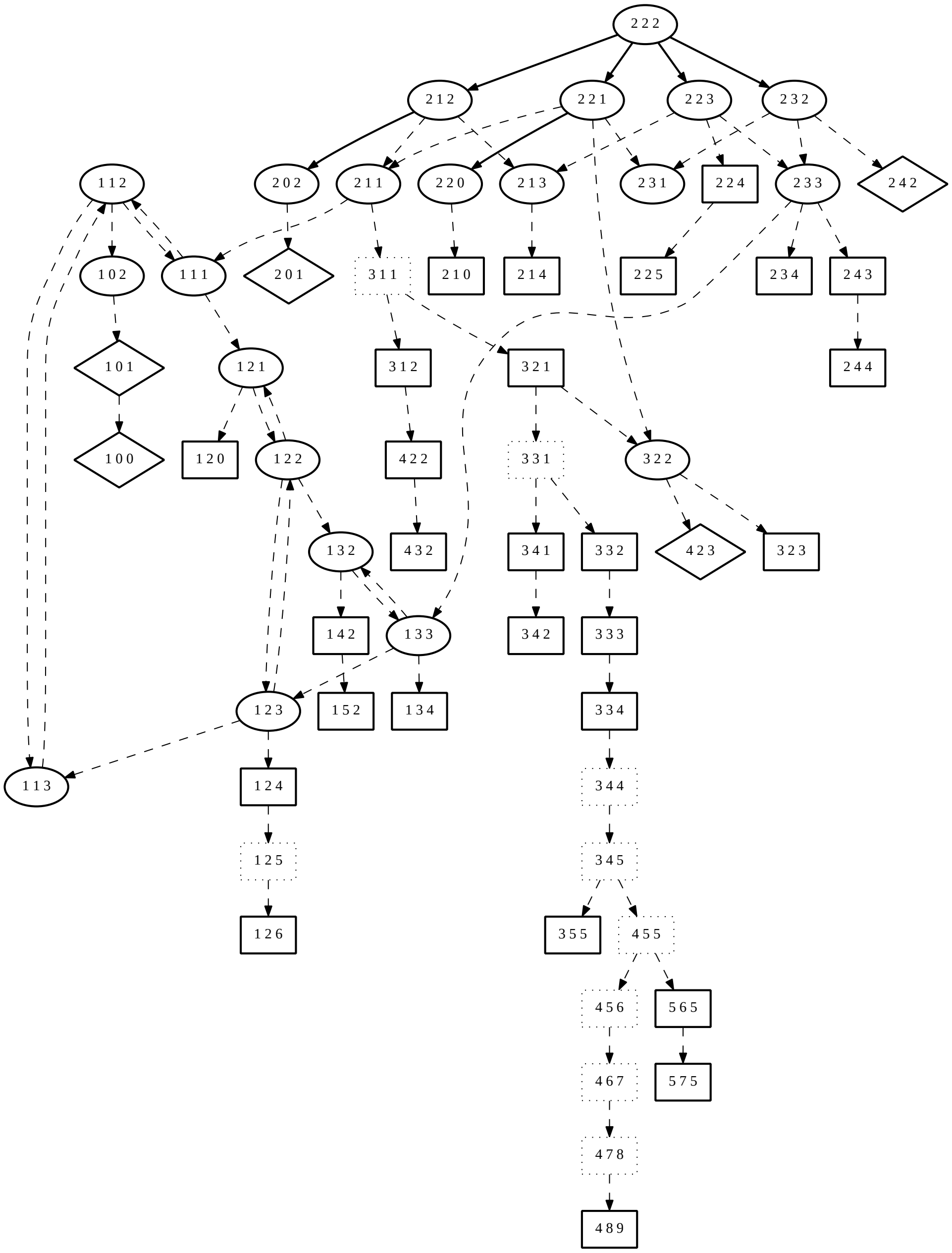

Comparison of Progression States via Consensus Graphs

As stated in the main manuscript, we considered three test statistics to evaluate the set of states that are shared between the paired samples, and we considered an unweighted and a weighted variant of each:

1. the fraction of observed nodes (cell count states) shared by the two trees;
2. the fraction of observed nodes shared by the two trees and reachable from the all-2 root
3. the fraction of observed nodes shared by the two trees and reachable via a shared path from the all-2 root

Each of the test statistics can be unweighted (count of the nodes) or weighted by the proportion of cells with each given cell count pattern. In either the count or the weighted form, each test statistic ranges between 0 and 1. For each of the weighted test statistics, the proportion of cells with some cell count pattern is defined as the arithmetic mean of the proportions in the two samples and their trees. For example, if a primary tumor tree has 20 observed states and the corresponding metastasis tree has 16 observed states and they share 8 states accounting for 60% (fraction 0.6) of the sampled cells in the primary sample and 80% (fraction 0.8) of the cells in the metastatic sample, then test statistic 2 would have value $0.5 \times ((8/20) + 8/16)) = 0.45$ and test statistic 3 would have value $0.5 \times (0.6 + 0.8) = 0.7$. Since we showed experimentally that all observed states are represented by tree nodes, test statistic 1 is the same whether computed from the input data directly or from the trees indirectly and does not take into account any evolutionary considerations. For these reasons, we focus attention on test statistics 2 and 3. In our implementation, test statistics 2 and 3 are computed as part of Algorithm S8, although this is not inherent to the algorithm. In general, higher values of the test statistic suggest that more of the evolution of the tumor took place in the common precursor of the two tumor states being compared, while lower values suggest that the two tumor states diverged early.

In both the main manuscript text and the following, we refer to test statistic 2 as "connected shared nodes" and test statistic 3 as "(nodes connected by) consensus path". For consistency with analyses in other sections, we computed single-sample merged trees on two gene probes at a time and the consensus networks for the trees with the same patient and the same two gene probes. Then, test statistics were collected either over all pairs of gene probes to get one set of values per patient or collected over all patients to get one set of values per gene pair. The per patient or per gene pair results are plotted in the box-and-whiskers style to show how the means vary across patients or gene pairs (on the x axis) and how big is the range of values (on the y axis) for one patient or one gene pair.

We applied the consensus graph method to evaluate similarity between primary and metastatic trees for individual CC patients. Fig. S2 shows the unweighted connected shared nodes (Fig. S2(a)), the weighted connected shared

Figure S2: **Statistics of similarity of CC trees between primary and metastastic plotted by patient.** Box-and-whisker plots show variability by gene pair for each patient. (a) Unweighted consensus shared node values. (b) Weighted consensus shared node values. (c) Unweighted consensus shared path values. (d) Weighted consensus shared path values.

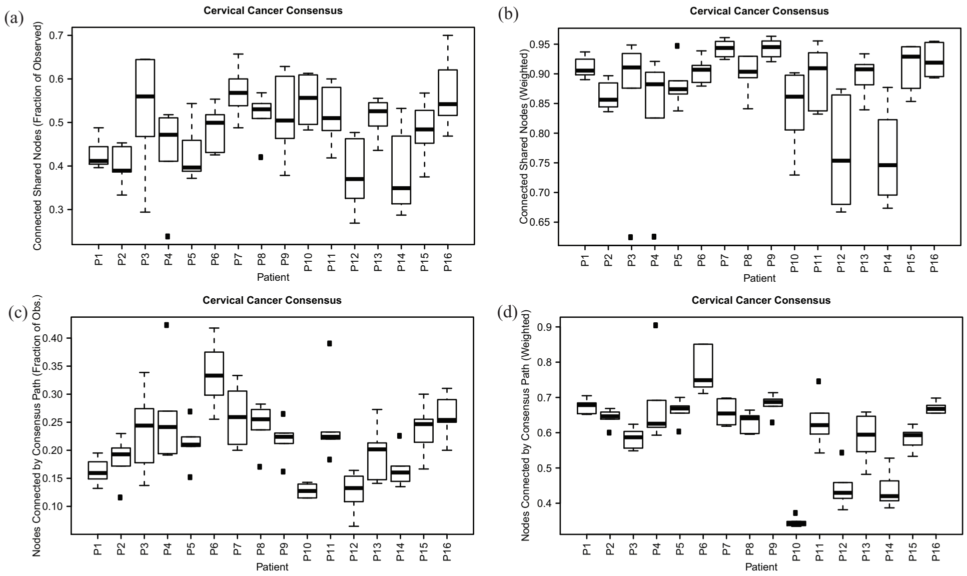

nodes (Fig. S2(b)), the unweighted consensus path (Fig. S2(c)), and the weighted consensus path (Fig. S2(d)) values per patient. In general, we observe that the values are low, suggesting that the primary tumor and metastasis diverged early from a common precursor. That is, the cells seeding the metastasis arise from clones generated early in the diversification of the primary tumor rather than from late stages in development of the primary.

However, there is considerable variation from one patient to another. For both statistics, the weighted values are higher than the unweighted values because the frequent states tend to be near the root and hence more likely to be in the common precursor. The consensus path values are substantially lower than the connected shared node values, suggesting that the two samples undergo some convergent evolution in which the same pattern of copy number changes arises multiple times in different orders. This observation of recurrent mutation represents a major difference from point mutations that are usually thought to occur exactly once in the evolution of any cancer, even across multiple samples from the patient. As was shown in the main paper, however, the values of weighted connected shared nodes are pretty similar across all six possible gene pairs for the weighted consensus path measure. The same is true for unweighted

Figure S3: **Statistics of similarity of BC trees between DCIS and IDC plotted by patient.** Box-and-whisker plots show variability by gene pair for each patient. (a) Unweighted consensus shared node values. (b) Weighted consensus shared node values. (c) Unweighted consensus shared path values. (d) Weighted consensus shared path values.

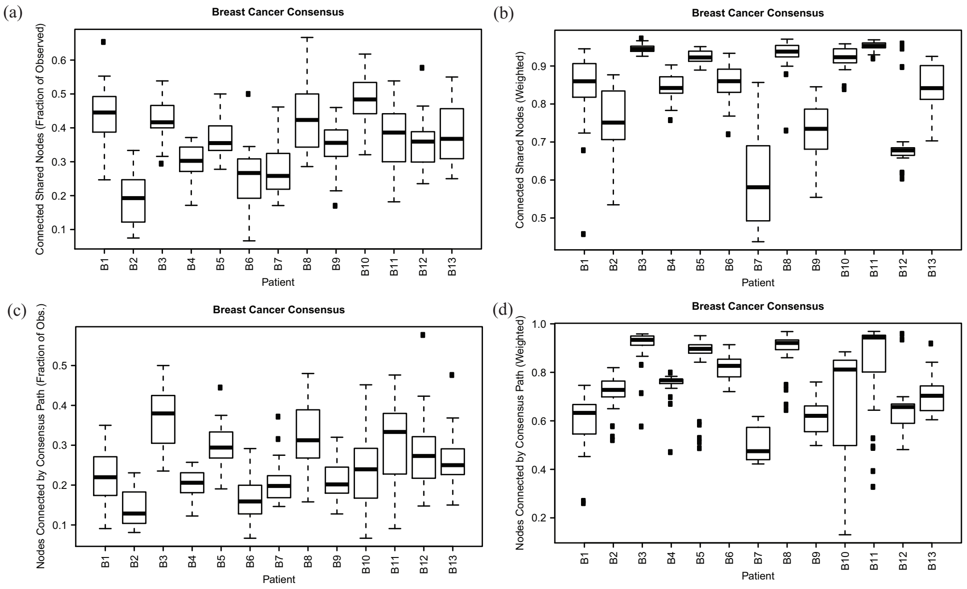

connected shared nodes, weighted shared nodes, and unweighted consensus path (figures not shown).

We also applied the consensus graph method to evaluate similarity between DCIS and IDC trees for individual BC patients. Fig. S3 shows the unweighted connected shared nodes (Fig. S3(a)), the weighted connected shared nodes (Fig. S3(b)), the unweighted consensus path (Fig. S3(c)), and the weighted consensus path values (Fig. S3(d)) per patient. As with the CC data set, we observe that the values are low, suggesting that the IDC arises from an early precursor of the DCIS and IDC, not by a linear progression from DCIS to IDC. This is against the conventional wisdom on the genomics of breast cancer progression [10] but consistent with emerging critiques of that consensus [11, 12]. As with the CC data set, the averages over gene pairs for each test statistic are fairly stable, as was shown in the main paper for the weighted consensus path statistic.

Table S1: Reconstruction error for FISHtrees without the monotonic ploidy rule, and for the ploidyless method. P-values were computed using a paired Wilcoxon rank-sum test. Test cases in which the ploidy-based solver failed to generate a tree were dropped entirely.

testset	ploidy-based		ploidy-less		
	mean	median	mean	median	P-value
Dataset 1	14.92	12.9	12.21	11.8	0.003794
Dataset 2	15.24	13.5	11.21	11.25	1.694e-05
Dataset 3	22.32	17.75	13.03	12.7	3.647e-11

Comparison with Monotonic Ploidy Rule Omitted

We tested the code with the monotonic ploidy rule omitted. Though the tree-merging problem remains feasible without this rule, in some instances, the resulting MILP is much more difficult to solve. For three of the 300 simulated trees, SCIP was unable to find a solution in eight hours on a modern (as of 2015) computer; by default FISHtrees terminates SCIP after 40 minutes, so small differences in hardware would not affect this observation. In contrast, SCIP never failed to find an answer when the monotonic ploidy rule was applied. The solution times without the monotonic ploidy rule are not universally long, but some instances appear to be exceptionally difficult.

For the simulated datasets, reconstruction error (Table S1) was worse, and the W statistic of parameter accuracy was not substantially different (data not shown). On the tumor datasets, omitting the monotonic ploidy rule did not make FISHtrees better at distinguishing early from advanced cancer, and in some cases made it worse. Indeed, the code without the monotonic ploidy rule could not distinguish DCIS from IDC by expected depth, or distinguish primary from metastatic CC by Shannon entropy or Simpson index (data not shown). Based on the theoretical arguments for using the monotonic ploidy rule, and the lack of empirical evidence that supports omitting it, we have enabled the monotonic ploidy rule in FISHtrees 3.0.

References

[1] Chowdhury SA, Shackney SE, Heselmeyer-Haddad K, Ried T, Schäffer AA, Schwartz R. Phylogenetic analysis of multiprobe fluorescence in situ hybridization data from tumor cell populations. Bioinformatics. 2013;29(13):i189–i198.

[2] Chowdhury SA, Shackney SE, Heselmeyer-Haddad K, Ried T, Schäffer AA, Schwartz R. Algorithms to Model Single Gene, Single Chromosome, and Whole Genome Copy Number Changes Jointly in Tumor Phylogenetics. PLoS Comp Biol. 2014;10(7):e1003740.

[3] Chowdhury SA, Gertz EM, Wangsa D, Heselmeyer-Haddad K, Ried T, Schäffer AA, et al. Phylogenetic analysis of multiprobe fluorescence in situ hybridization data from tumor cell populations. Bioinformatics. 2013;31(12):i258–i267.

[4] Gerlinger M, Rowan AJ, Horswell S, Larkin J, Endesfelder D, Gronroos E, et al. Intratumor heterogeneity and branched evolution revealed by multiregion sequencing. N Engl J Med. 2012;366(10):883–892.

[5] Sottoriva A, Spiteri I, Piccirillo SG, Touloumis A, Collins VP, Marioni JC, et al. Intratumor heterogeneity in human glioblastoma reflects cancer evolutionary dynamics. Proc Natl Acad Sci USA. 2010;110(10):4009–4014.

[6] Almendro V, Cheng Y, Randles A, Itzkovitz S, Marusyk A, Amettler E, et al. Inference of tumor evolution during chemotherapy by computational modeling and in situ analysis of genetic and phenotypic cellular diversity. Cell Rep. 2014;6(3):514–527.

[7] Pennington G, Smith CA, Shackney S, Schwartz R. Reconstructing tumor phylogenies from heterogeneous single-cell data. J Bioinform Comput Biol. 2007;5(2a):407–427.

[8] Cormen TH, Leiserson CE, Rivest RL. Introduction to Algorithms. MIT Press and McGraw-Hill; 1990.

[9] Fletcher R. Practical Methods of Optimization. Wiley; 1987.

[10] Klein CA. Selection and adaptation during metastatic cancer progression. Nature. 2013;501:365–372.

[11] Polyak K. Is breast tumor progression really linear? Clin Cancer Res. 2008;14(2):339–341.

[12] Carraro DC, Elias EV, Andrade VP. Ductal carcinoma in situ of the breast: morphological and molecular features implicated in progression. Biosci Rep. 2014;34(1):18–27.
